# Bioprinted Tumor Microenvironment Models Reveal Immune Evasion and Guide CAR‐NK Therapeutic Strategies

**DOI:** 10.1002/advs.202521188

**Published:** 2026-05-08

**Authors:** Dahong Kim, Seona Jo, In‐Hwan Jang, Yu‐Jin Kim, Youngmee Jung, Junhyoung Ahn, Hyungjun Lim, Jae Jong Lee, Kangwon Lee, Tae‐Don Kim, Su A Park

**Affiliations:** ^1^ Nano Lithography & Manufacturing Research Center Nano‐Convergence Manufacturing Research Division Korea Institute of Machinery and Materials (KIMM) Daejeon Republic of Korea; ^2^ Department of Applied Bioengineering Graduate School of Convergence Science and Technology Seoul National University Seoul Republic of Korea; ^3^ Center for Gene and Cell Therapy Korea Research Institute of Bioscience and Biotechnology (KRIBB) Daejeon Republic of Korea; ^4^ KRIBB School of Advanced Bioconvergence University of Science and Technology (UST) Daejeon Republic of Korea; ^5^ Center for Biomaterials Korea Institute of Science and Technology Seoul Republic of Korea; ^6^ Department of Electrical and Electronic Engineering YU‐KIST Institute Yonsei University Seoul Republic of Korea; ^7^ Department of Pharmacy Yonsei Institute of Pharmaceutical Sciences Department of Integrative Biotechnology College of Pharmacy Yonsei University Incheon Republic of Korea

**Keywords:** 3D bioprinting, CAR‐NK cell therapy, immune‐tumor interaction, immunosuppression, immunotherapy, tumor microenvironment (TME)

## Abstract

The clinical outcome predictions of conventional in vitro and in vivo models are often inaccurate because they cannot replicate the tumor microenvironment (TME) complexity. Existing 3D models encounter challenges regarding TME complexity replication, engineering constraints, and limited capacity in analyzing immune‐cancer interactions. This study employs 3D embedded bioprinting to develop a heterogeneous lung spheroid (HLS) model, incorporating key stromal factors to better reflect the TME. Transcriptomic profiling via RNA sequencing reveals gene signatures associated with extracellular matrix remodeling, immune suppression, and tumor progression, demonstrating substantial similarity to patient‐derived lung tumor samples and validating the biological fidelity of the model. Functional assays demonstrate that the model effectively replicated TME dynamics, as evidenced by reduced CAR‐NK cell infiltration, cytotoxicity, and cytokine secretion with increasing model complexity, indicative of a highly immunosuppressive environment. Advanced CAR‐NK cells expressing chemokine receptors are utilized to overcome this immune barrier and enhance migration and infiltration within the physiologically relevant lung TME model. Overall, this model replicates critical features of the lung TME, showing potential for evaluating next‐generation immunotherapies targeting complex solid tumors.

## Graphical abstract

This study presents a 3D embedded bioprinting platform that recapitulates key stromal features of the tumor microenvironment using fibroblasts and lung‐derived ECM. The model enables functional assessment of CAR‐NK cells and provides a versatile tool to support the development of next‐generation immunotherapeutic strategies against solid tumors.

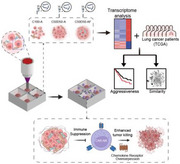

## 1 Introduction

Preclinical in vitro and in vivo models, including immunotherapies, are essential for developing and evaluating therapeutic strategies for solid tumors. However, these models often fail to capture the full complexity of the tumor microenvironment (TME), limiting their predictive value for clinical translation. Conventional 2D models fail to replicate the spatial and biochemical complexity of living tissues. In vivo models, particularly immunodeficient mice, poorly mimic human immune responses due to interspecies differences, limiting their clinical relevance [1, 2, 3, 4]. Consequently, promising cell‐based immunotherapies such as chimeric antigen receptor (CAR)‐T and CAR‐NK show efficacy in preclinical studies but fail in clinical trials due to the lack of physiologically relevant models that accurately reproduce immune‐tumor interactions [5, 6]. Therefore, developing advanced TME platforms is essential for replicating tumor architecture, stromal complexity, and immunosuppressive signaling to accurately simulate immune dynamics in solid tumors [4, 7, 8, 9, 10].

A physiologically relevant TME model must incorporate intrinsic characteristics (genetic and epigenetic alterations driving clonal evolution) and extrinsic stromal components (cancer‐associated fibroblasts (CAFs), immune cells, and the extracellular matrix (ECM) [11, 12]. These components collectively contribute to tumor progression and regulate immune cell responses [13, 14, 15, 16]. Effective modeling of immune‐tumor‐stromal interactions requires advanced platforms capable of reproducing the spatial and functional complexity of the TME under controlled conditions [17, 18].

3D bioprinting is a promising approach for constructing physiologically relevant TME models by enabling the precise spatial organization of multiple cell types and ECM components [19, 20]. These capabilities facilitate the development of customizable in vitro models that capture tumor heterogeneity, immune cell evasion, and stromal interactions, thereby advancing immunotherapy research [21, 22].

Despite significant progress, current 3D bioprinted models often fall short in reproducing the full immunological complexity of the TME. Techniques such as hanging‐drop and microfluidic systems face scalability, structural complexity, and immunological environmental control limitations. Moreover, many 3D bioprinted models lack diverse immune cell populations and are ineffective for conducting mechanistic immune suppression and cytotoxicity investigations. Therefore, there is a critical need for next‐generation in vitro platforms that mimic the architectural and molecular features of the TME and enable systematic investigation of immune‐tumor interactions under physiologically relevant conditions [7, 8, 9, 10].

**FIGURE 1:**
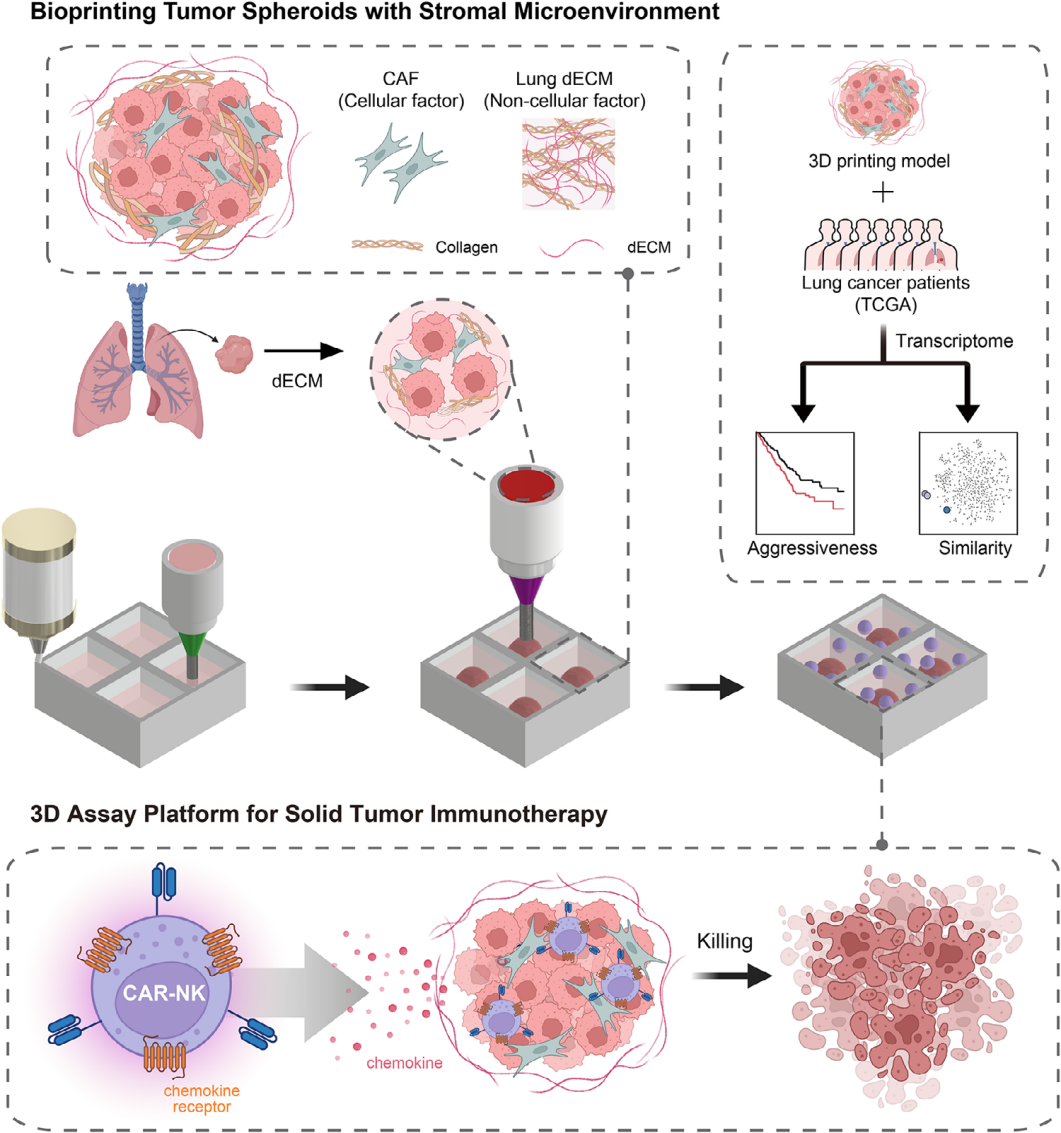
Schematic representation of the 3D bioprinting process. Schematic diagram of the 3D bioprinting process to fabricate a heterogeneous lung cancer spheroid model with cellular and non‐cellular factors. Depiction of NK cell application targeting EGFR (cancer‐specific antigen) and CXCR6 chemoreceptors, investigating immune‐tumor interactions within an immunosuppressive environment.

This study employed 3D embedded printing to model tumor heterogeneity and examine immune‐tumor‐stromal cell interactions, establishing an immunotherapy testing platform (Figure 1). Non‐small cell lung cancer (NSCLC), the predominant subtype of lung cancer, was selected as a representative disease model due to its clinical significance and poor prognosis [23, 24, 25]. Bioinks were formulated to integrate both cellular (CAFs) and non‐cellular (collagen, decellularized lung‐derived ECM (dECM) components, improving tumor fidelity. Transcriptomic analysis revealed strong alignment between the printed model and lung cancer patient samples, supporting its biological relevance for investigating tumor progression and immune regulation. The heterogeneous lung tumor spheroid (HLS) model was integrated with epidermal growth factor receptor (EGFR) CAR‐NK cells to assess immune suppression functionally. HLS suppressed CAR‐NK cytotoxicity and cytokine secretion, reflecting the immunosuppressive role of the TME. To address the immunosuppressive nature of solid tumors, advanced CAR‐NK cells were engineered to express chemokine receptors, leveraging a physiologically relevant lung TME model enriched with tumor‐specific chemokine cues.

This 3D bioprinting platform effectively mimics the lung cancer microenvironment, providing a robust tool for studying immune‐tumor dynamics and optimizing immunotherapeutic strategies, with strong potential for enhancing preclinical immunotherapy testing and clinical translation.

## 2 Results

### 2.1 Hydrogel Characterization Using dECM for 3D Bioprinted Tumor Models

**FIGURE 2:**
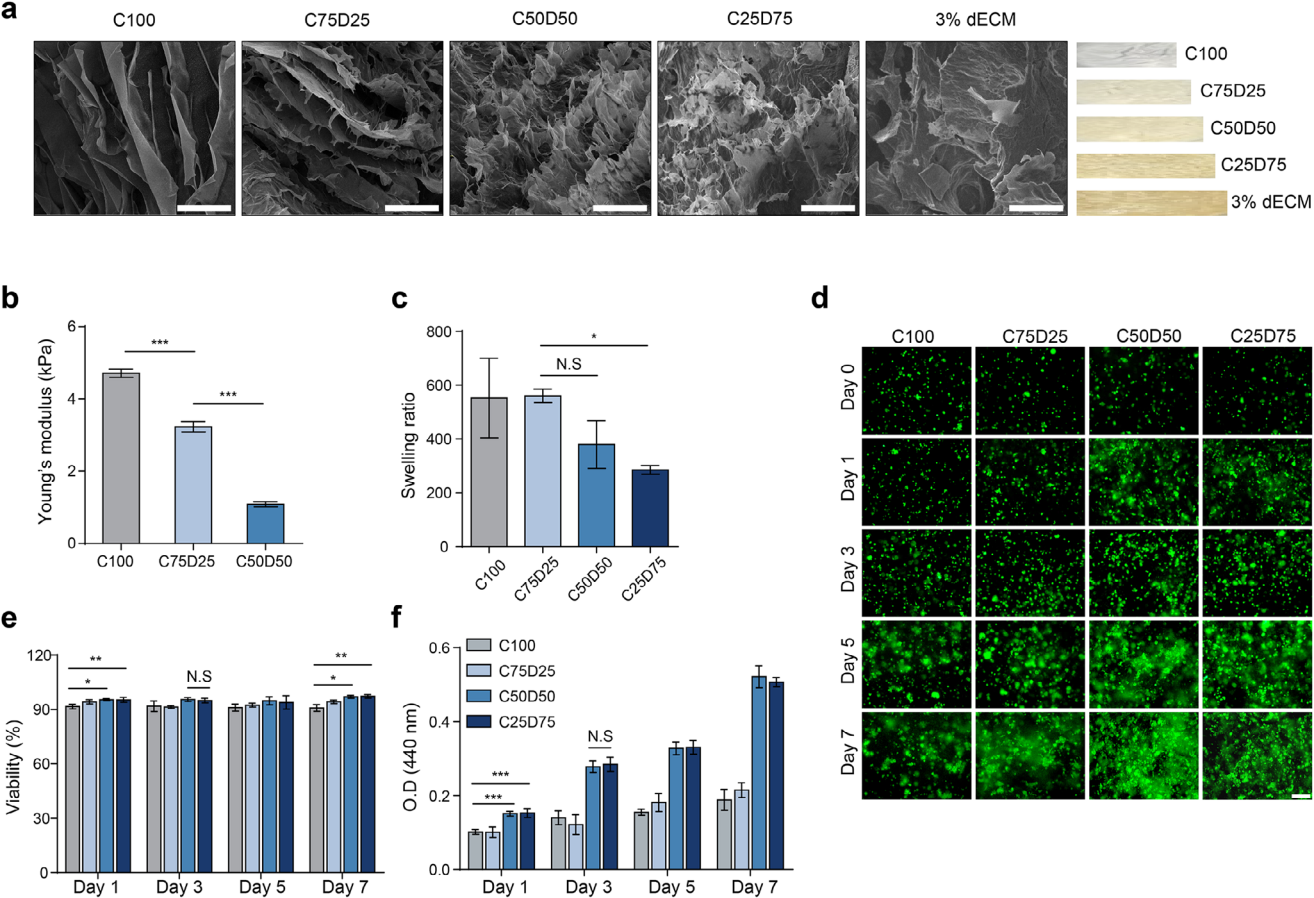
Characterization of dECM‐based hydrogels. (a) Representative SEM image showing the pore morphology and microscopic image demonstrating the color change in the bio‐ink composed of collagen and 3% dECM at varying ratios; scale bar: 200 µm. (b) Young's modulus of bio‐ink. (c) Swelling ratio of bio‐ink. Data are mean ± s.d. (*n* = 3). Statistical significance was determined using one‐way ANOVA. (d) Representative fluorescent images depicting live and dead assays for the A549 cell line encapsulated in bio‐ink over a period from 0 to 7 days; scale bar: 500 µm. (e) Quantification of cell viability in bio‐ink, measured by the ratio of the A549 cell area to total cell area. (f) Quantification of cell proliferation of tumor cells encapsulated in bio‐ink. Data are mean ± s.d. (*n* = 5). Statistical significance was determined using one‐way ANOVA.

To replicate the biochemical and structural features of native lung tissue, lung‐derived dECM was employed as a stromal component in bioink formulations. The decellularization process was critical for removing residual DNA, minimizing immunogenicity, and enhancing suitability for 3D bioprinting [26, 27, 28]. To fabricate hydrogels suitable for human lung spheroid (HLS) printing, bioinks were prepared with varying ratios of 1% collagen and 3% dECM: 100:0 (C100), 75:25 (C75D25), 50:50 (C50D50), and 25:75 (C25D75). Scanning electron microscopy (SEM) analysis revealed distinct alterations in hydrogel morphology with increasing dECM content (Figure 2a). Incorporation of dECM altered the fibrillar morphology of the hydrogels, resulting in a more disordered network and a pronounced yellowish hue.

Mechanical characterization revealed that C100, C75D25, and C50D50 exhibited Young's moduli within the reported physiological range of lung tissue (1–5 kPa) [29, 30, 31], measuring 4.71 ± 0.12, 3.23 ± 0.15, and 1.09 ± 0.07 kPa, respectively (Figure 2b). However, C25D75 demonstrated excessive fluidity, precluding accurate modulus measurement. Healthy human lung parenchyma typically exhibits stiffness in the ∼1–3 kPa range [32], whereas lung tumors and fibrotic regions generally demonstrate increased stiffness depending on stage and stromal remodeling, with reported values ranging from 2 kPa to over 20 kPa [33]. The measured values approximate the mechanical properties of soft or early‐stage lung tumor microenvironments.

Swelling, an essential factor influencing nutrient diffusion and cell‐matrix interactions, was also assessed (Figure 2c). A gradual reduction in swelling ratio was observed with increasing dECM content: C100 at 552.41%, C75D25 at 560.64%, C50D50 at 379.49%, and C25D75 at 284.88%. These findings suggest that dECM‐rich hydrogels have reduced water retention capacity, potentially affecting long‐term matrix stability and cellular behavior. The biocompatibility of the bioinks was assessed through live/dead staining and WST‐1 assays. All groups maintained cell viability above 90% during 7 days of culture (Figure 2d,e), confirming the biocompatibility of the dECM‐based formulations. Notably, cell proliferation patterns varied with dECM content. While the C100 and C75D25 groups exhibited modest increases in proliferation over time, the C50D50 and C25D75 groups exhibited significantly enhanced proliferation (Figure 2f). No statistically significant difference was observed between C50D50 and C25D75, suggesting a saturation point beyond which additional dECM does not further enhance proliferation.

### 2.2 Optimizing Bio‐Ink and Printing Bath for Fabricating Precise Tumor Spheroid

**FIGURE 3:**
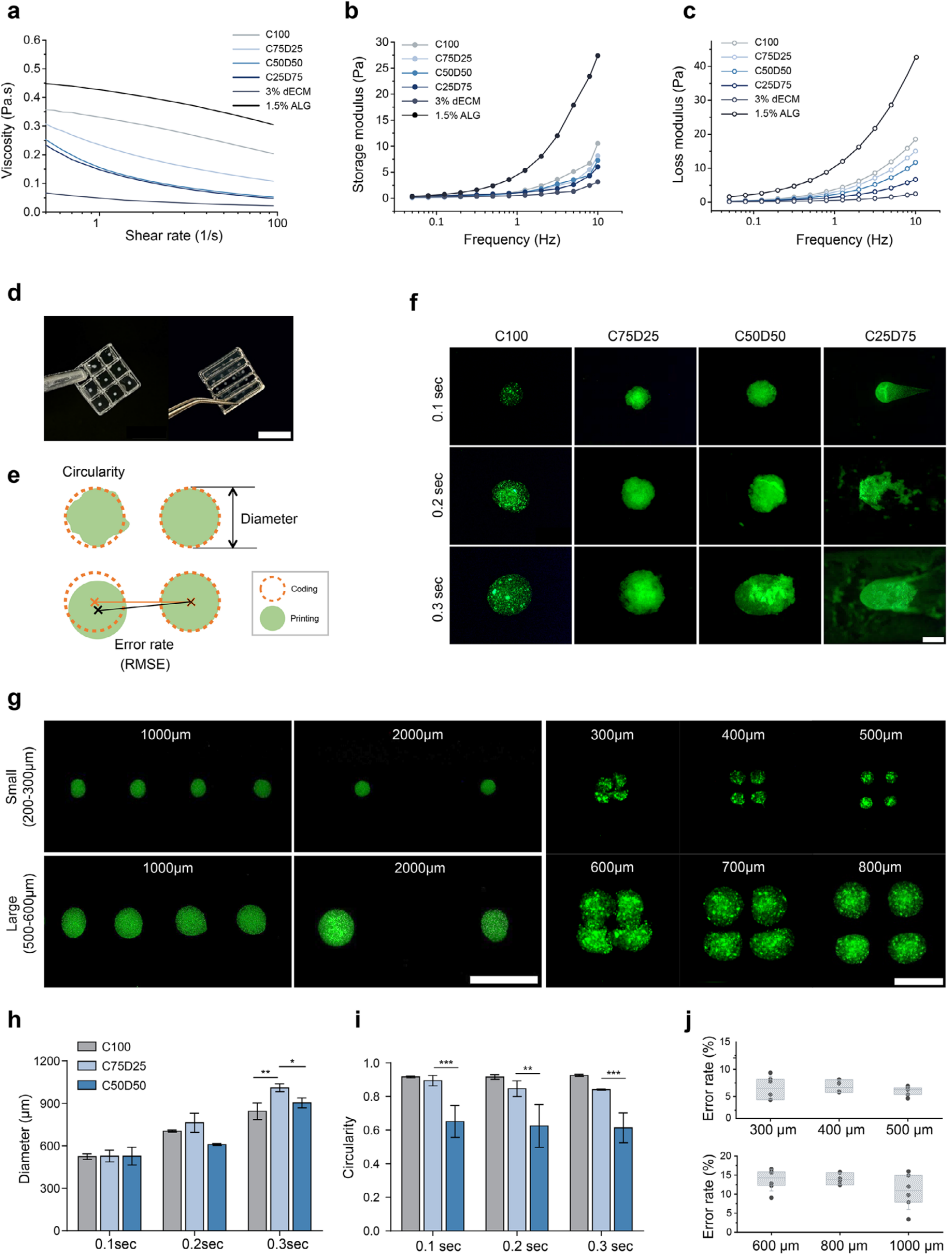
Optimization of bio‐ink and printing bath. (a) Viscosity characteristics of bio‐ink and printing bath. (b) Storage modulus and (c) loss modulus measurements for the bio‐ink and printing bath. ALG indicates alginate. (d) Representative image of a printed EVA chip, demonstrating adjustments in the structure and positioning of the printed spheroids; scale bar: 10 mm. (e) Schematic illustration of the measurement process for diameter, circularity, and error rate of printed spheroid. (f) Evaluation of printability and spheroid size by adjusting the printing pressure and the ratio of collagen to dECM in the bio‐ink; scale bar: 500 µm. (g) Representative fluorescent images depicting the printable distance for spheroids sized small (200–300 µm) and large (500–600 µm). Right panel: reduced inter‐center spacing associated with compromised spheroid integrity and separation.; scale bar: 1000 µm. (h) Quantification of spheroid size and (i) circularity of spheroids. Sec indicates the printing time (seconds). Data are mean ± s.d (*n* = 4). (j) Quantification of the error rate between the actual printing position and the coded position using the root mean square error (RMSE). Data are mean ± s.d, with the interquartile range from the 25th to 75th percentile (*n* = 6). Statistical significance was determined using one‐way ANOVA.

To achieve structurally and biologically viable HLS constructs, collagen‐dECM bioinks were used in a bath‐based embedded printing approach. The printed ethylene vinyl acetate (EVA) chip was used to support the alginate bath and stabilize the printed HLS (Data S1). The rheological properties of the bio‐ink were characterized to evaluate its viscoelastic behavior, which is critical for maintaining structural integrity during and after the printing process. All formulations exhibited shear‐thinning behavior, with viscosity decreasing with increasing shear rate, facilitating smooth extrusion and rapid recovery to preserve structural fidelity (Figure 3a). This viscoelastic recovery also minimizes shear‐induced cell damage, supporting high cell viability throughout the printing process [34, 35]. Storage and loss moduli measurements indicated that the loss modulus was consistently higher than the storage modulus across all groups, especially in dECM‐rich bioinks, reflecting a more fluid‐like behavior (Figure 3b,c). A hydrogel‐supported bath printing approach was employed to address the challenges of low structural stability associated with high fluidity, in which alginate served as a low‐viscosity support bath to maintain the printed structure during fabrication. Rheological analysis demonstrated that alginate exhibited higher storage modulus and viscosity than the hydrogels used as bioinks, supporting its role as a stabilizing matrix during the printing process. Printability was evaluated based on shape fidelity (circularity), size control, and deposition accuracy (error rate) (Figure 3e). Most formulations maintained circularity values above 0.5. However, higher dECM content was associated with reduced shape retention (Figure 3f,i). The size of the printed constructs was modulated by adjusting pneumatic pressure, producing structures ranging from 500 to 1000 µm in diameter (Figure 3f,h). Deposition accuracy was assessed by calculating the root mean square error (RMSE) between coded and actual positions. Deposition accuracy was assessed by calculating the root mean square error (RMSE) between coded and actual spheroid positions. The upper panel corresponds to smaller printed spheroids (approximately 200–300 µm in diameter), whereas the lower panel represents larger spheroids (approximately 500–600 µm in diameter), consistent with the size regimes shown in Figure 3g. Larger printed structures exhibited higher positional error compared to smaller spheroids, while increasing inter‐center spacing tended to reduce the mean positional error. Accordingly, higher precision was achieved with smaller printed structures and wider spacing. These results indicate that positional accuracy remained stable across the tested range, with error rates below 20% in all groups, while still reflecting size‐dependent printing behavior. (Figure 3g,j).

While bio‐inks containing 3% dECM demonstrated favorable printability, 1% dECM formulations showed poor structural retention despite supporting high cell viability (Data S3). Based on the combined evaluation of printability and cellular proliferation, the C50D50 formulation was selected for subsequent experiments, offering balanced mechanical performance and biological compatibility optimized for HLS fabrication with a targeted diameter of 500 µm.

### 2.3 Comparison of Morphological and Cellular Changes in 3D Tumor Models with Increasing TME Complexity

**FIGURE 4:**
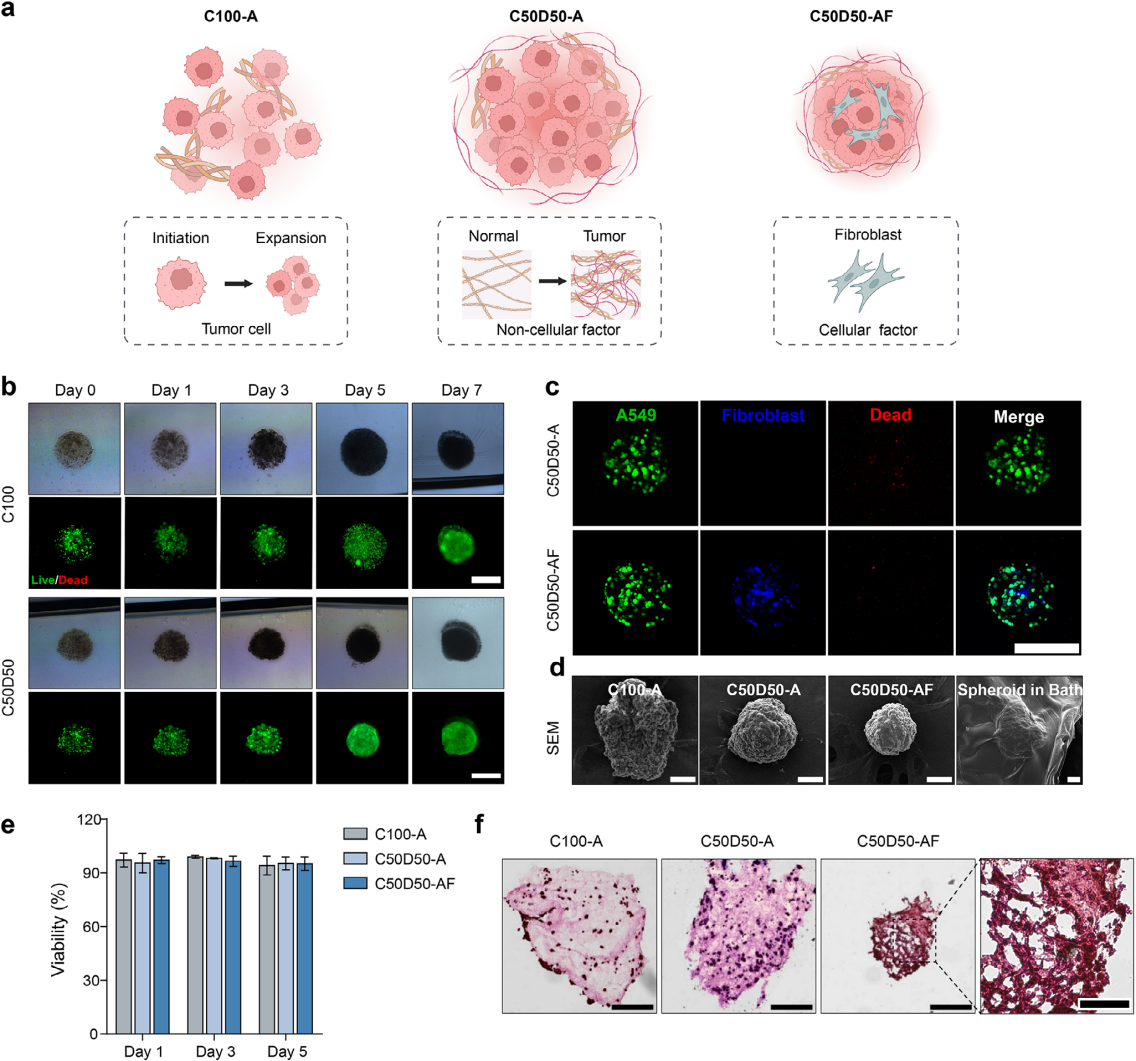
Morphological and cellular characterization of HLS model. (a) Schematic illustration of depicting the intrinsic and extrinsic factors in the C100‐A, C50D50‐A, and C50D50‐AF models. (b) Representative microscopic and fluorescent images of printed tumor spheroids, depicting conditions with and without dECM; Red: dead cells; Green: live cells; scale bar: 500 µm. (c) Representative confocal images showing the presence or absence of fibroblasts in C50D50 bioink; Green: live cells; Blue: human lung fibroblasts; Red: dead cells; scale bar: 500 µm. (d) Representative SEM images: C100‐A, C50D50‐A (with dECM), and C50D50‐AF (with dECM and lung fibroblast) bioinks; scale bar: 100 µm. (e) Cell viability within the bio‐ink was quantified by the A549 cell area to total cell area ratio. Data are mean ± s.d (*n* = 5). Statistical significance was determined using one‐way ANOVA. (f) Representative H&E‐stained images of C100‐A, C50D50‐A, and C50D50‐AF (scale bar: 250 µm), and a magnified view of the C50D50‐AF (scale bar: 100 µm), showing histological differences.

The printed HLS incorporated cellular and non‐cellular components to accurately mimic the tumor microenvironment (Figure 4a). The non‐cellular component, dECM, was adjusted to create a suitable environment for HLS. For comparison, bio‐inks were prepared using C100 hydrogel loaded with A549 cells, a non‐small cell lung cancer line (C100‐A), and C50D50 hydrogel loaded with A549 cells (C50D50‐A) (Figure 4b). Additionally, to evaluate the relationship between A549 cells and stromal cells, human lung fibroblasts (HLFs) were added to the C50D50 hydrogel in a 2:1 ratio with A549 cells to create C50D50‐AF bio‐ink (Figure 4c). All spheroids were printed under conditions that yielded structures with a diameter of approximately 500 µm. Across all groups, cell viability remained consistently high, exceeding 90% throughout the five‐day culture period, with no significant changes observed (Figure 4e). Cell density increased over time, as indicated by the progressive rise in fluorescence intensity from GFP. Notably, HLFs in the C50D50‐AF group displayed uniform distribution throughout the spheroids by Day 3.

The printed spheroids exhibited distinct morphological characteristics. Spheroid size progressively decreased, and structural compactness increased across the C100‐A, C50D50‐A, and C50D50‐AF conditions. Incorporation of dECM reduced the storage modulus of the bioink and was associated with increased cellular proliferation, collectively contributing to spheroid contraction (Figure 4d). Furthermore, the printed spheroid exhibited a well‐defined circular morphology in the side view (Data S5c). These trends were also confirmed through H&E staining (Figure 4f). Among the groups, the C100‐A group displayed the lowest cell density and the largest spheroid size. However, the C50D50‐AF group demonstrated comparatively dense cellular structures, with abundant cytoplasm and prominent intercellular bridges. This phenomenon resembles the characteristics commonly observed in pulmonary fibrosis and NSCLC tissues [36, 37, 38].

### 2.4 Transcriptomic Analysis of the 3D Lung Tumor Model and the Functional Similarity of its Results to Lung Tumor Patient Data

**FIGURE 5:**
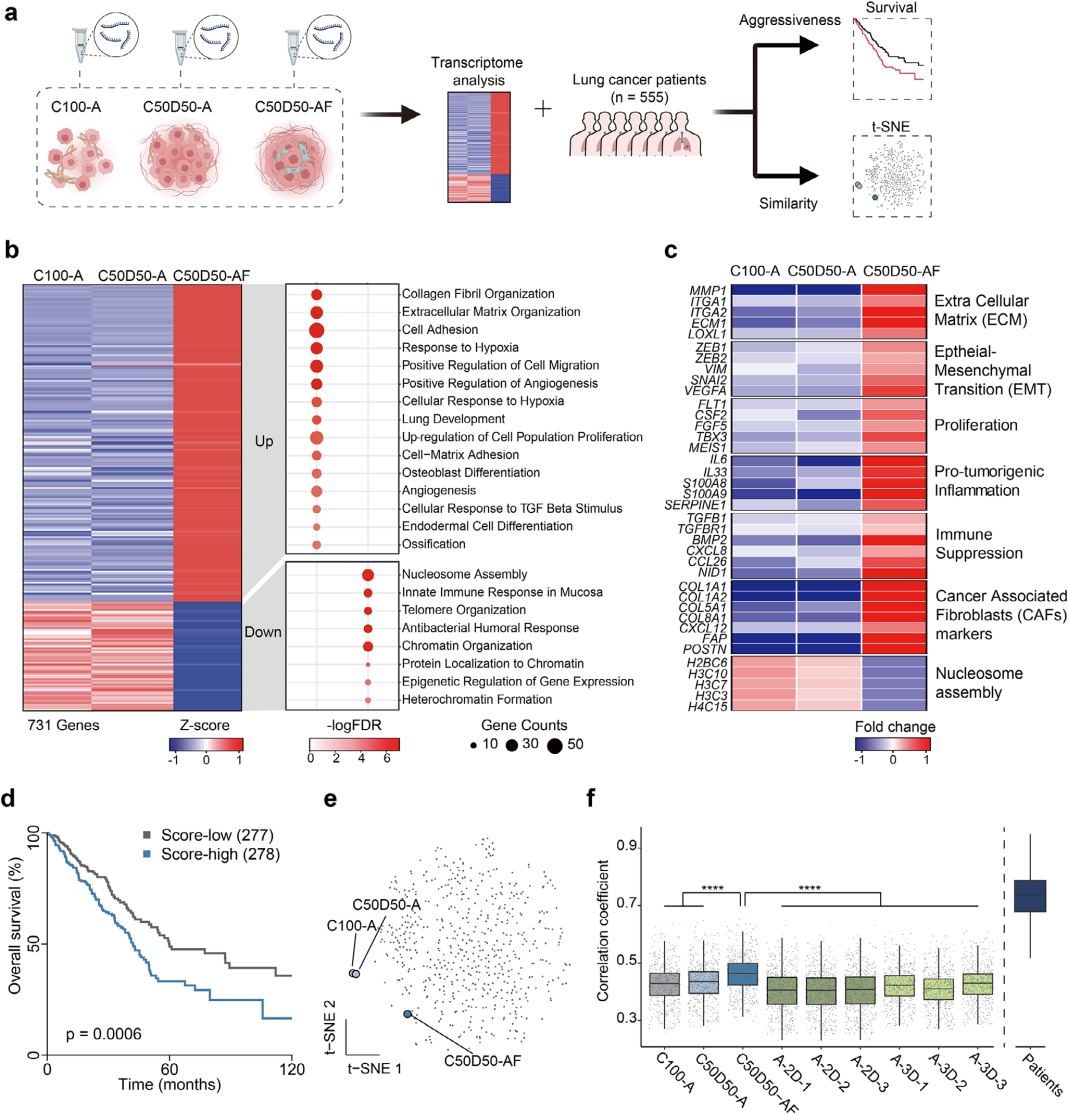
RNA sequencing validation of a heterogeneous tumor microenvironment in the 3D lung tumor model. (a) Workflow of transcriptome analysis and clinical data integration to evaluate tumor aggressiveness and similarity to patient tumors. (b, c) Transcriptome analysis of the C100‐A, C50D50‐A, and C50D50‐AF models. (b) Left: heatmap of 731 differentially expressed genes (DEGs) between C50D50‐AF and the other two models (fold change > 1.5). Color scale indicating high (red) and low (navy) expression levels. Right: gene ontology (GO) enrichment analysis of biological process for upregulated (up) and downregulated (down) regulated genes (FDR < 0.01). Symbol size represents the number of genes associated with each term. Color scale indicating FDR significance (low FDR; red). (c) Gene expression patterns associated with functional changes for the HLS model. Color scale indicating high (red) and low (navy) expression levels. (d) Kaplan–Meier curve of two groups in TCGA patients with lung cancer (*n* = 555) stratifies by the gene set of differentially expressed in C50D50‐AF. Score‐high and score‐low patients; high/low expression of C50D50‐AF upregulated genes, and low/high expression of downregulated genes, respectively. Statistical significance was determined using the log‐rank test. (e) t‐SNE projection of HLS model (large dots) associated gene set from TCGA patients with lung cancer (*n* = 555; small dots) and three models (C100‐A, C50D50‐A, and C50D50‐AF) expression levels. (f) Distribution of correlation coefficients compared to patient samples. Grey dots indicate correlation with each patient's samples. A‐2D‐1, ‐2, and ‐3 represent 2D cultures with A549 cells from GSE130590. A‐3D‐1, ‐2, and ‐3 represent 3D cultures with the same cells and dataset. Patients indicating TCGA lung cancer patients. Statistical significance was calculated by Analysis of Variance (ANOVA, ^∗∗∗∗^ *p* < 0.0001). All transcriptomic analyses were performed using R software (version 4.2.1).

To evaluate the functional relevance of the 3D‐printed HLS models to patient tumors, transcriptomic profiles of the three different models (C100‐A, C50D50‐A, and C50D50‐AF) were analyzed and integrated with public lung cancer datasets (Figure 5a). To identify differentially expressed genes (DEGs) while ensuring an adequate number of genes for a robust and meaningful Gene Ontology (GO) enrichment analysis, we employed a 1.5‐fold change threshold. By comparing the C50D50‐AF model against the other two models, we identified a specific HLS‐mediated gene set consisting of 731 DEGs to explore TME complexity, survival outcome, and transcriptomic similarity to patient tumors. Gene enrichment analysis revealed that genes significantly upregulated in the C50D50‐AF model were associated with collagen fibril and extracellular matrix organization (MMP1, ITGA1, ITGA2, ECM1, and LOXL1), indicating increased TME complexity and functional fibrosis (Figure 5b,c). Additionally, genes related to epithelial‐mesenchymal transition (EMT; ZEB1, ZEB2, VIM, SNAI2, and VEGFA), cell proliferation (FLT1, CSF2, FGF5, TBX3, and MEIS1), pro‐tumorigenic inflammation (IL6, IL33, S100A8, S100A9, and SERPINE1), and immune suppression (TGFB1, TGFBR1, BMP2, CXCL8, CCL26, and NID1) were also upregulated. Furthermore, CAF marker genes (COL1A1, COL1A2, CXCL12, FAP, and POSTN) were significantly upregulated, supporting the acquisition of CAF‐like transcriptional features within the structured HLS model. However, genes involved in nucleosome assembly, particularly histone proteins, were significantly downregulated, suggesting a shift in chromatin structure and transcriptional regulation. These findings suggest that a structured HLS model promotes tumor progression, metastasis, and immune evasion.

To further validate these functional changes, the relationship between the gene set derived from the C50D50‐AF sample and tumor samples from lung cancer patients was evaluated (Figure 5c). Lung cancer patients with higher scores based on this gene set exhibited significantly poorer prognoses than those with lower scores, reflecting the enrichment of genes associated with tumor progression, metastasis, and immune evasion (Figure 5d). Additionally, t‐SNE analysis revealed that the C50D50‐AF sample clustered with patient‐derived expression data, distinguishing it from the less advanced 3D models (C100‐A and C50D50‐A) (Figure 5e). Further transcriptome‐wide correlation analysis, including comparisons with other datasets of Matrigel‐based 3D cultures derived from A549 cells, showed that C50D50‐AF exhibited a significantly higher correlation with patient samples (mean correlation *r* = 0.46) compared to the other groups (*r* ≈ 0.40–0.43). However, a numerical gap remains when compared to the high inter‐patient correlations observed within the TCGA dataset (mean *r* > 0.70) (Figure 5f). Despite this difference, these findings collectively demonstrate that the printed HLS more effectively reproduces the functional aspects of tumor progression, fibrosis, and immune suppression compared to existing models. Consequently, strong transcriptomic similarity with aggressive lung tumors is observed, supporting the use of HLS as a robust in vitro model for investigating tumor aggressiveness and immune evasion.

### 2.5 Immunosuppressive Effects of 3D Tumor Models on CAR‐NK Cell Efficacy

The HLS models were used to evaluate the response of EGFR CAR‐NK cells. The NSCLC cell line, A549, known to overexpress the tumor‐associated antigen EGFR, served as the target (Data S4a,b). In preliminary 2D in vitro co‐culture assays, EGFR CAR‐NK cells demonstrated significantly enhanced efficacy compared to conventional NK92 cells. Specifically, they exhibited a five‐fold increase in calcein‐AM‐based cytotoxicity at an effector‐to‐target (E:T) ratio of 5:1 and a two‐fold increase in apoptosis induction at a 1:1 E:T ratio (Data S4c). These findings validated the use of EGFR CAR‐NK cells for assessing immune responses within the HLS model.

**FIGURE 6:**
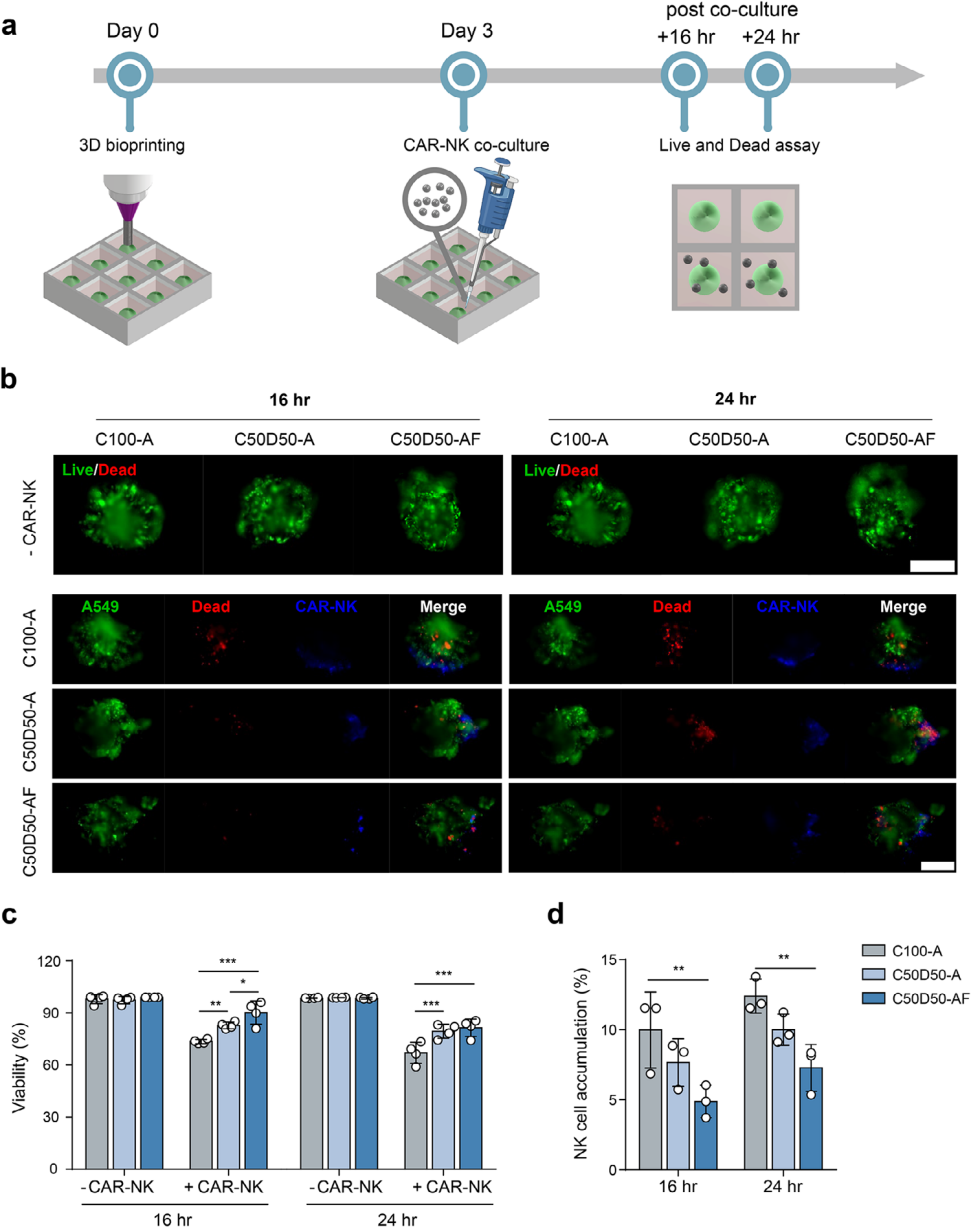
Evaluation of CAR‐NK cell cytotoxicity and recruitment in the HLS model. (a) Schematic diagram illustrating the experimental setup, including the HLS culture period, loading of cells, and reaction times. (b) Representative fluorescent images of 3D‐printed HLS models co‐cultured with CAR‐NK cells for 16 and 24 h. Red: dead cells; Green: live cells; Blue: NK or CAR‐NK cells; scale bar: 250 µm. (c) Tumor cell viability in the presence and absence of CAR‐NK cells (*n* = 4). ‘−CAR NK’ represents the target‐only group (no NK cells added). (d) Quantification of NK cell accumulation near target cells in the HLS models, expressed as the NK cell area to total cell area ratio (*n* = 3). Data are mean ± s.d. Statistical significance was determined using one‐way ANOVA.

Printed HLS models were cultured for 3 days to allow TME maturation. Subsequently, EGFR CAR‐NK cells were introduced on the bath surface over each well containing the HLS models, and immune responses were assessed during 16 and 24‐h co‐culture periods (Figure 6a,b). To evaluate CAR‐NK‐mediated tumor killing and immune cell recruitment, we compared these responses across HLS models containing different stromal components, including C100‐A, C50D50‐A, and C50D50‐AF.

In the absence of EGFR CAR‐NK cells, viability exceeded 95% across all groups (Figure 6c). However, tumor cell viability was significantly higher in the C50D50‐AF group compared to the C100‐A and C50D50‐A groups following CAR‐NK co‐culture, indicating reduced CAR‐NK‐mediated cytotoxicity in this condition. While prolonged co‐culture with CAR‐NK cells generally reduced the viability of A549 cells, the C50D50‐AF group, representing the high‐complex HLS condition, exhibited a slower reduction in viability (Figure 6b,c; Data S4d,e). These findings suggest that the inclusion of stromal components, such as fibroblasts and dECM, may suppress immune responses induced by CAR‐NK cells. The recruitment of immune cells was also evaluated (Figure 6d). Over time, all groups exhibited an increasing trend in CAR‐NK cell recruitment to the HLS model periphery. However, recruitment was significantly lower in the C50D50‐AF group, which mimics the TME more closely [39, 40].

### 2.6 Functional Alterations in Tumor and CAR‐NK Cells with Increasing TME Complexity

**FIGURE 7:**
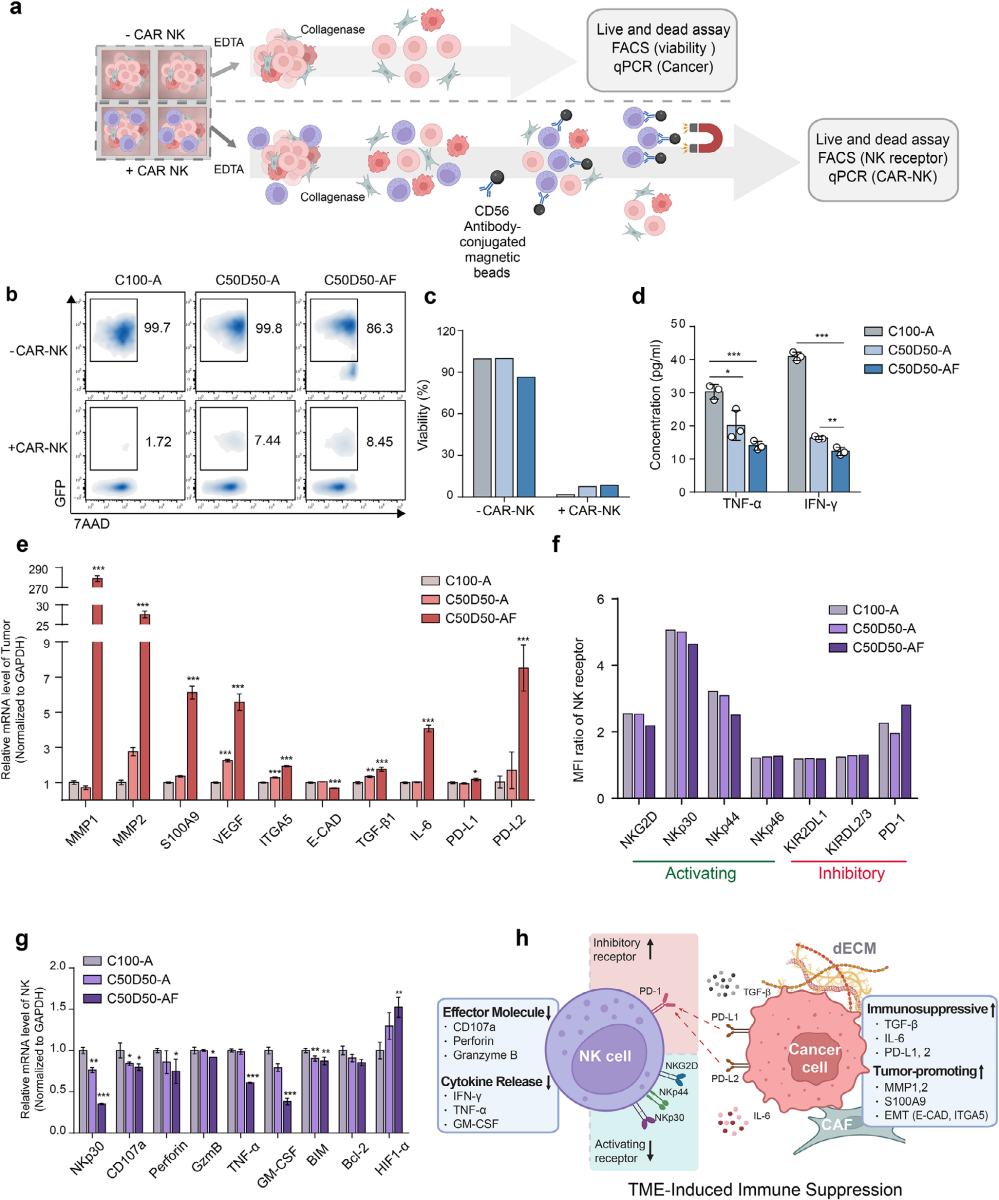
Quantitative analysis of tumor‐immune interactions in HLS constructs. (a) Schematic diagram of the experiments conducted with or without CAR‐NK from a 3D printed HLS model. (b) Flow cytometric analysis of tumor cells (GFP+) dissociated after co‐culture with CAR‐NK cells. (c) Quantification of live tumor cells (GFP+7AAD‐). (d) human TNF‐α and IFN‐γ released by CAR‐NK cells co‐cultured in the HLS model. Data are mean ± s.d. (*n* = 3). (e) mRNA expression levels in tumor cells are isolated from the 3D printed model. Data are mean ± s.d. (*n* = 3). (f) Flow cytometric analysis of surface receptors on CAR‐NK cells isolated after co‐culture in the HLS model. g. mRNA expression levels in CAR‐NK cells are isolated after co‐culture in the HLS model. Data are mean ± s.d. (*n* = 3). Statistical significance was determined using one‐way ANOVA. h. Schematic illustration of gene‐level alterations in NK and cancer cells under immunosuppressive TME.

Following the initial quantitative analysis via fluorescence imaging, FACS analysis was subsequently performed to provide a more precise assessment of cancer cell viability and status (Figure 7a–c). The tumor cells and recruited immune cells within the HLS model were dissociated using EDTA and collagenase at concentrations that do not compromise cell viability (Data S6). A comparison of viable cancer cell populations revealed that 8.45% of the cells remained viable in the high‐complexity group (C50D50‐AF) compared to 1.72% in the low‐complexity group (C100‐A). A similar trend was observed in the cytokine release assay (Figure 7d). These results suggest that a TME‐mimicking model diminishes the anti‐cancer efficacy of CAR‐NK cells.

Cancer and immune cells were analyzed to investigate the mechanisms underlying the reduced anti‐cancer efficacy in HLS models that better mimic the TME. To separate cancer and CAR‐NK cells, NK cells were positively selected using CD56 microbeads (Figure 7a; Data S7). Results from qPCR and FACS analysis revealed that in high‐complexity HLS models, cancer cells exhibited upregulation of genes involved in extracellular matrix remodeling (MMP1, MMP2), inflammation (S100A9), angiogenesis and metastasis (VEGF, ITGA5, E‐cadherin), and immune suppression (TGF‐β, IL‐6, PD‐L1, PD‐L2) (Figure 7e) [41, 42]. These expression changes are consistent with the broader patterns observed in the global transcriptomic analysis (Figure 5). In contrast, the expression of NK activating receptors (NKG2D, NKp30, and NKp44), cytolytic‐related genes (CD107a, Perforin, and Granzyme B), and inflammatory cytokines (GM‐CSF and TNF‐α) was reduced as the HLS model complexity increased (Figure 7f,g) [43, 44, 45].

Together, these results show that increasing HLS complexity is associated with tumor induction of immunosuppressive/checkpoint signals (TGF‐β/IL‐6, PD‐L1/PD‐L2) and concurrent reduction of CAR‐NK effector programs, consistent with enhanced immune evasion (Figure 7h). These findings highlight that the HLS models can serve as a functional platform for evaluating the therapeutic limitations imposed by the TME and underscore the importance of developing enhanced CAR‐NK strategies to overcome immunosuppressive barriers [46, 47].

### 2.7 Enhanced Recruitment and Antitumor Activity of CXCR6 + CAR‐NK Cells in an Immunosuppressive 3D Tumor Model

To effectively target solid tumors, we focused on chemokine receptors due to their critical role in guiding immune cell migration and infiltration into the TME. Chemokines and their receptors have been widely recognized as key regulators in overcoming the physical and immunosuppressive barriers of solid tumors [48, 49, 50, 51, 52].

**FIGURE 8:**
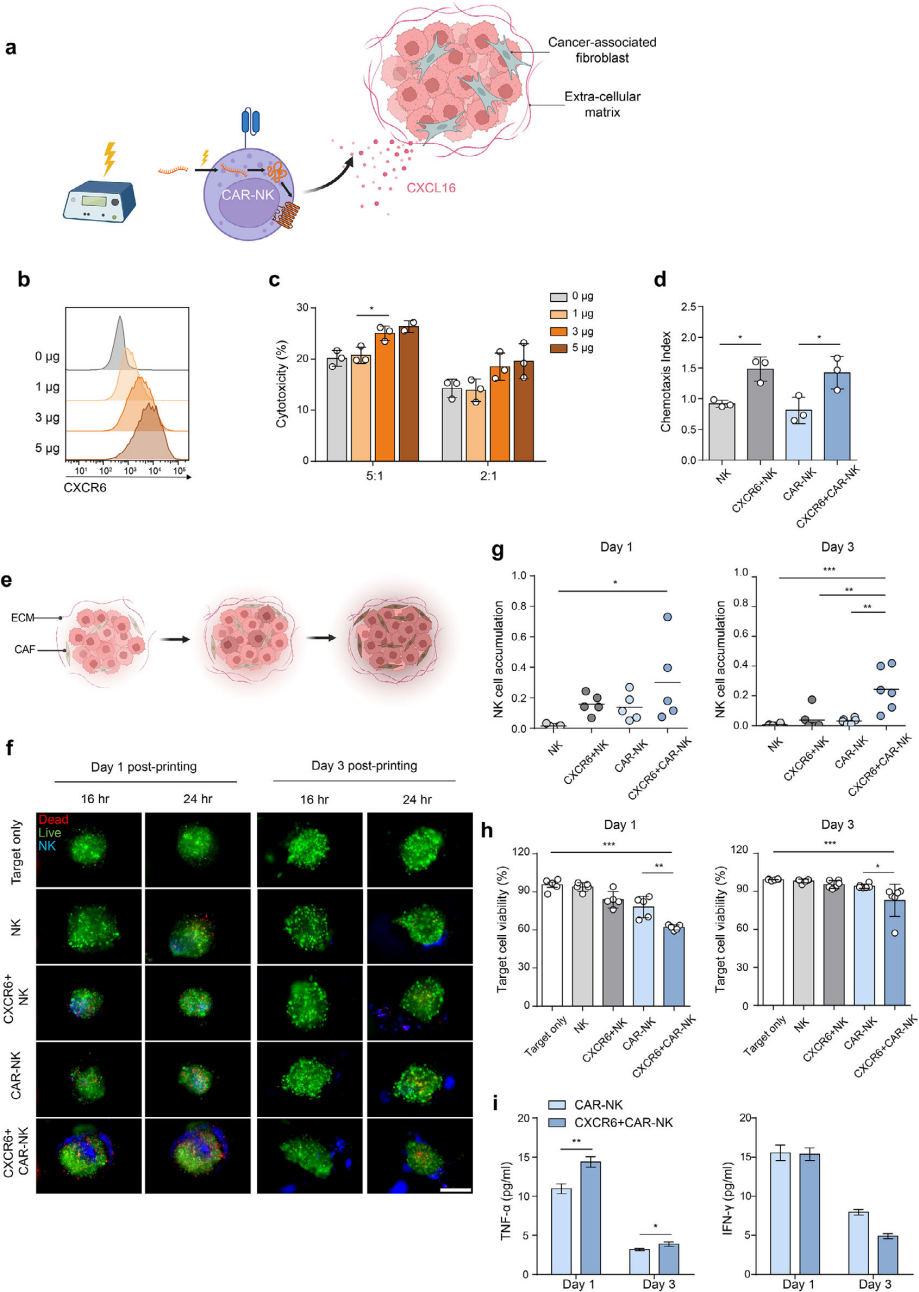
Evaluation of CXCR6+CAR‐NK cell recruitment and cytotoxicity in a CXCL16‐enriched 3D lung tumor model. (a) Diagram illustrating CXCR6 mRNA transfection into CAR‐NK cells via electroporation and the interaction between CXCR6+ CAR‐NK cells and CXCL16 secreted by the H460‐based HLS model. (b) CXCR6 expression was analyzed by flow cytometry across varying mRNA concentrations. (c) Cytotoxicity of NK cells against H460 cells based on the level of CXCR6 expression. (d) Migration of NK and CAR‐NK cells toward 50 ng/µL CXCL16 was evaluated using a transwell assay after 4 h of incubation, based on CXCR6 expression. (e) Schematic representation of tumor model progression over time following 3D printing. (f) Representative fluorescent images of NK or CAR‐NK cells with and without CXCR6 co‐cultured in the H460‐based HLS model at Days 1 and 3 post‐printing. Red: dead cells; Green: live cells; Blue: NK or CAR‐NK cells. (g) NK cell accumulation near target cells in the HLS model, quantified as the NK cell area/total cell area ratio at Days 1 and 3 post‐printing. (h) Target cell viability analysis after 24 h of co‐culture in the HLS model at Days 1 and 3 post‐printing. Data are mean ± s.d. (*n* = 5, 6). Statistical significance was determined using two‐way ANOVA. i. Human TNF‐α and IFN‐γ released by CAR‐NK cells with CXCR6 co‐cultured in the HLS model. Data are mean ± s.d. (*n* = 3). Statistical significance was determined using one‐way ANOVA.

To identify chemokines enriched in lung tumors, the mRNA levels of CXC‐chemokines in the NSCLC cell line H460 were analyzed (Data S8). CXCL3 and CXCL16 exhibited high expression among the chemokines. However, this study focused on CXCL16 due to its functional advantage in NK cell recruitment [53]. Specifically, its receptor CXCR6 is highly expressed in NK cells enriched in the lung, which is believed to influence NK cell migration and adhesion to the lung [54, 55, 56]. Furthermore, CXCL16 expression was also observed in other lung cancer cell lines, supporting its relevance across multiple NSCLC models (Data S8b). Electroporation‐based mRNA transfection of NK cells resulted in a dose‐dependent increase in CXCR6 expression (Figure 8a,b). To verify functional CXCR6 signaling following mRNA transfection, we assessed CXCL16‐induced phosphorylation of ERK and AKT, canonical downstream readouts of the CXCR6–CXCL16 axis [57, 58]. Time‐course analysis demonstrated ligand‐responsive phosphorylation ≥6 h post‐transfection (Data S9a,b), and subsequent experiments were performed accordingly. In the 2D functional assay, the 5 ug group exhibited 1.3‐fold higher cytotoxicity compared to the 0 ug control group (Figure 8c). The transwell migration assay showed a 1.75‐fold increase in migration toward CXCL16 in the CXCR6 overexpression group (CXCR6+NK, CXCR6+CAR‐NK) (Figure 8d). Based on these results, CXCR6 overexpression in CAR‐NK cells significantly enhanced both migration and anticancer efficacy. The improved functionality of CXCR6‐expressing immune cells was further tested using the HLS model.

For this HLS model, the NSCLS cell line H460 was used because of its high CXCL16 expression, enabling evaluation of CXCR6‐overexpressing NK cells. As tumors evolve, increased ECM deposition and CAF accumulation restrict immune cell infiltration and promote immune resistance (Figure 8e). Consistent with this, co‐incubation in the Day 3 HLS model showed a substantial reduction in cell accumulation and cytotoxicity compared to the Day 1 model (Figure 8f–h). Despite these barriers, the CXCR6+ CAR‐NK group demonstrated a 7.7‐fold increase in NK cell accumulation at the tumor periphery (Figure 8g) and an 11.15% reduction in target cell viability relative to the CAR‐NK group (Figure 8h). This enhanced antitumor response was further supported by increased TNF‐α secretion in the co‐culture supernatants (Figure 8i), confirming that CXCR6 expression improves NK cell trafficking and function even under immunosuppressive 3D tumor conditions. A similar experiment was conducted using a model without dECM (Data S10). In this model, NK cell accumulation in the CXCR6+ CAR‐NK group increased by 14.04‐fold (Data S10c), while target cell viability decreased by 6.92% compared to the CAR‐NK group (Data S10d). Notably, CAR‐NK cell accumulation and cytotoxicity were higher in the model without dECM, suggesting that the HLS model incorporating fibroblasts and dECM more accurately mimics the immunosuppressive TME.

These findings demonstrate that the HLS model replicates critical features of tumor progression, while CXCR6 expression enhances NK cell migration and cytotoxicity within an immunosuppressive environment, suggesting a promising strategy for overcoming TME barriers.

## 3 Discussion

This study established a 3D embedded bioprinting model to effectively mimic the tumor heterogeneity and the immune microenvironment of lung cancer. By integrating key stromal factors such as CAFs and dECM, the model recreated the complexity of the TME and enabled in‐depth analysis of interactions with CAR‐NK cells. The findings demonstrated that increased TME complexity activates immunosuppressive mechanisms that inhibit CAR‐NK cell infiltration, cytotoxicity, and cytokine secretion. Furthermore, the model facilitated the evaluation of advanced CAR‐NK cells engineered with lung cancer‐specific chemokine receptors, highlighting its utility for probing diverse modes of immune–tumor interactions.

The study emphasizes several innovations compared to previous approaches. 3D embedded printing enables precise spheroid positioning and consistent sample production, supported by a customized chip with a compartmentalized structure that minimizes external variability during immune interaction studies. Collectively, these design features position the HLS model as a controlled and standardized platform to dissect stromal and ECM‐driven constraints on NK‐based immunotherapies under defined conditions. Furthermore, tailoring the mechanical environment to mimic soft tissue properties enhances cellular responsiveness and model fidelity [59, 60, 61, 62].

While most 3D bioprinting studies focus on morphological and phenotypic analyses, this study notably incorporates transcriptomic analysis to assess the functional fidelity of the bioprinted tumor constructs. Integration with patient‐derived transcriptomic profiles revealed that the HLS model enriched genes associated with ECM remodeling, EMT, invasive phenotype, and immune evasion, all contributing to poor prognosis in lung cancer patients [63, 64, 65, 66]. Notably, the C50D50‐AF condition exhibited the highest transcriptomic similarity to patient tumor samples, supporting its relevance as a physiologically representative in vitro tumor model. Enrichment of canonical CAF‐associated gene signatures supports fibroblast activation toward a CAF‐like transcriptional state within the structured HLS model. Further protein‐level and functional validation would allow more precise characterization of fibroblast activation in this system. Compared with other models, the improved transcriptomic similarity between C50D50‐AF and lung tumor patient samples the ability of the HLS model, which highlights its potential as a robust in vitro platform for studying tumor progression and immune evasion.

Moreover, this study is the first to systematically analyze EGFR CAR‐NK cell behavior within a 3D tumor model, revealing key mechanisms underlying immune suppression. The HLS model successfully captured critical features of the immunosuppressive TME, including matrix remodeling and pro‐tumor signaling. These changes were accompanied by the functional inhibition of CAR‐NK cells, evidenced by downregulation of activating receptors and effector molecules. These findings highlight the importance of incorporating stromal, biochemical, and immune components into tumor models to guide the development of next‐generation immunotherapies.

To overcome TME‐induced suppression of CAR‐NK efficacy, we explored advanced therapeutic strategies to enhance immune cell infiltration and cytotoxicity in solid tumors. We demonstrated that engineering CAR‐NK cells to express the chemokine receptor CXCR6 significantly enhanced their migration and antitumor activity within the HLS model. Importantly, this benefit should be interpreted in the context of a CXCL16‐positive tumor microenvironment, as CXCL16 expression can vary across tumor types and patient subsets. More broadly, chemokine‐guided CAR‐NK design may be best implemented as a personalized approach, in which chemokine receptor selection is tailored to tumor‐ and patient‐specific chemokine landscapes. Together, these findings expand the utility of the HLS platform as a framework for evaluating context‐specific chemokine‐driven immune responses.

Despite a significant advancement, certain limitations remain. Although the incorporation of fibroblasts and dECM improved transcriptomic alignment with patient tumors, further protein‐level and functional validation would enable more precise characterization of fibroblast activation and stromal remodeling. In addition, correlation with patient data remains lower than that among patient samples, supporting the need to incorporate patient‐derived materials (PDO‐ or PDX‐derived cells) to better capture patient‐specific TME complexity and genetic diversity. This discrepancy likely reflects underrepresented features of the native tumor microenvironment, including diverse immune cell populations, hypoxic gradients, and patient‐specific stromal variability. Accordingly, the immunosuppressive phenotype modeled here primarily reflects stromal (CAF/dECM)‐driven constraints, and conclusions regarding CAR‐NK performance should be interpreted within this defined context. Therefore, future work should focus on modeling the tumor‐immune microenvironment (TIME), including additional immune populations such as regulatory T cells and tumor‐associated macrophages. Reconstructing the TIME will allow a more comprehensive evaluation of immunotherapy efficacy and resistance mechanisms [67, 68]. Furthermore, integrating functional vascular features will be essential to more accurately recapitulate oxygen and nutrient gradients that regulate hypoxia‐driven signaling, angiogenic remodeling, metastatic dissemination, and immune cell trafficking within the tumor microenvironment [69, 70]. Overall, this 3D bioprinting platform represents a substantial advancement for studying tumor–immune interactions and evaluating cancer immunotherapies. While additional engineering optimization will be necessary to enable large‐scale implementation, it establishes a foundation for translational immunotherapy screening using reproducible bioprinted tumor models. The ability to generate physiologically relevant and structurally defined tumor microenvironments holds strong potential for improving preclinical assessments and bridging the gap toward clinical translation.

## 4 Conclusion

In this study, we established a 3D embedded bioprinting platform that replicates the cellular complexity of TME by incorporating stromal components such as fibroblasts and lung‐derived dECM. This model enabled systematic investigation of immune–tumor interactions under biologically relevant conditions. The HLS model exhibited gene expression patterns comparable to those of patient‐derived lung tumor tissues, particularly in pathways associated with tumor progression and immune evasion, supporting its biological fidelity and translational potential. CAR‐NK cell functionality—including infiltration, cytotoxicity, cytokine secretion, and activation marker expression—was progressively diminished with increasing TME complexity. To overcome this immunosuppressive environment, CAR‐NK cells were engineered to express the chemokine receptor CXCR6, which enhanced their tumor infiltration and antitumor efficacy within the model. This chemokine‐guided strategy underscores the potential of immune cell engineering to improve the performance of CAR‐based therapies in solid tumors.

Altogether, our findings demonstrate that the HLS model serves as a versatile platform for investigating tumor–immune dynamics and advancing the development of next‐generation immunotherapies targeting solid tumors.

## 5 Experimental Section/Methods

### 5.1 Bioink Preparation

A bioink hydrogel was prepared using 1% collagen (MS bio) and dECM from the lung of a porcine. The dECM was provided by KIST (Korea Institute of Science and Technology, South Korea). dECM was prepared by dissolving in 0.5 m acetic acid containing 3 mg/mL pepsin (Roche, Switzerland) and neutralized using NaOH and MEM (Sigma–Aldrich, USA) at a final concentration of 3% dECM. Bioink hydrogels were prepared with 1% collagen and 3% dECM in a specific volume ratio (100:0, 75:25, 50:50, 25:75, 0:100) and stirred homogeneously. Cells were immediately embedded into the prepared hydrogel. Tumor cells were embedded at a density of 1 × 10^7^ cells/mL. Fibroblasts were included at 5 × 10^6^ cells/mL, corresponding to a tumor‐to‐fibroblast ratio of 2:1.

### 5.2 Circularity and Deposition Accuracy

A bioink hydrogel was prepared at defined volume ratios and printed under a constant pressure of 15 kPa, while varying the printing time, which refers to the duration of pneumatic pressure applied to the syringe. Circularity was calculated as follows:

$$\mathrm{Circularity}=4\pi A/L^{2}$$

where *A* is the area of the printed structure, and *L* is the perimeter. A circularity value of 1 corresponds to a perfect circle.

Deposition accuracy was quantified using root mean square error (RMSE). A reference dot was selected, and the spatial deviations of the four adjacent printed dots relative to their predefined positions were used for error calculation.

### 5.3 3D Cell Printing

HLS was fabricated with bioink by using the bath printing method. EVA chip, which could support the hydrogel bath and spheroid, was fabricated using a bioprinter with a 400‐µm‐diameter nozzle under a pressure of 350 kPa. Each well of the EVA chip was filled with 20 µL of 1.5% (w/v) alginate prepared in PBS at room temperature. HLS printing was performed under different time conditions and printing positions to produce the desired dot shape and size (Figure 3d–j). HLS was printed at 15 kPa for 0.1 s, resulting in a spheroid diameter of approximately 500–600 µm. Following printing, constructs were crosslinked by immersion in 3% CaCl_2_ solution for 3 min and subsequently washed with PBS.

### 5.4 Bioink and Cell Morphology

The bioink morphology and that of the spheroid after HLS printing were confirmed with SEM. Bioink with a specific volume ratio was prepared by placing it in a cylindrical mold with an 8‐mm diameter for sampling and then freeze‐drying. The dried hydrogel was put into liquid N_2_ for over 1 min and cracked quickly to examine the cross‐section. To investigate the bioink morphology and that of the cells after HLS printing, scanning electron microscopy (SEM) was used. The ink, prepared at a specific volume ratio, was placed in a cylindrical mold with an 8‐mm diameter and then freeze‐dried. The freeze‐dried hydrogel was briefly immersed in liquid nitrogen and fractured to observe its cross‐section.

For HLS constructs, post‐printing samples with cell components were fixed in 4% paraformaldehyde for more than 24 h. The fixed samples were dehydrated through a graded ethanol series with increasing concentrations (10%, 30%, 50%, 70%, 90%, and 100%). Finally, the samples were sputter‐coated with platinum (Pt) under the conditions of 30 mA for 120 s. Then, SEM imaging was performed to examine their morphology.

### 5.5 Mechanical and Rheological Properties

A universal testing machine (EZ test; SHIMADZU, Japan) was used to evaluate the Young's modulus of the bioink. A plate jig with a diameter of 6 mm was used, and the test speed was set to 2 mm/min to ensure accurate mechanical property measurements. A rotational rheometer was used to evaluate the rheological properties of the bioink. A parallel plate with a diameter of 40 mm and a gap of 1000 µm was used at an oscillation amplitude of 0.01%, frequency of 1 Hz, and room temperature.

### 5.6 Cell Culture

The non‐small cell lung cell line (A549, H460, PC‐9, H1650) was cultured in Roswell Park Memorial Institute (RPMI; Welgen, South Korea) medium 1640 containing 10% fetal bovine serum (FBS; R&D Systems, USA) and 1% antibiotic–antimycotic (Gibco, USA). The human lung fibroblast was cultured in DMEM (Gibco) with 10% FBS and 1% penicillin. NK92 (human natural killer cell line; CRL‐2407TM) cells and EGFR CAR‐NK92 cells were cultured in alpha‐MEM (Welgene, South Korea) supplemented with 0.2 mm inositol, 0.1 mm 2‐mercaptoethanol, 0.02 mm folic acid (Sigma–Aldrich, USA), 12.5% fetal bovine serum (FBS) (R&D systems, USA), 12.5% fetal horse serum (Gibco, USA), 1% antibiotic–antimycotic and 200 IU/mL human recombinant IL‐2 (#200‐02, PeproTech, USA). EGFR‐targeting CAR‐NK cells used in this study were previously established in our laboratory [71, 72]. Detailed CAR generation procedures have been described previously [73].

All cells were cultured in 5% CO_2_ at 37°C. A549 (ATCC, CCL‐185; RRID:CVCL_0023), H460 (ATCC, HTB‐177; RRID:CVCL_0459), PC‐9 (ECACC, 90071810; RRID:CVCL_B260), H1650 (ATCC, CRL‐5883; RRID:CVCL_1483), NK92 (ATCC, CRL‐2407; RRID:CVCL_2142) and primary human lung fibroblasts (ATCC, PCS‐201‐013; RRID: N.A.) were obtained from the indicated repositories. All cell lines were confirmed mycoplasma‐negative using a commercial detection kit (#25239, iNtRON Biotechnology, Korea) prior to experiments.

### 5.7 Cell Viability and Proliferation

A live/dead assay (#L3224, Thermo Fisher Scientific, USA) was used to evaluate the cell viability and spheroid formation. Calcein–AM and ethidium homodimer‐1 were added to PBS for the staining solution following the manufacturer's instructions. The samples were washed with PBS and stained for 1 h. The fluorescence was investigated using a fluorescence microscope (Eclipse Ti, Nikon, Japan). To measure cell proliferation in bioink and estimate the number of cells in HLS, the Premix WST‐1 Cell Proliferation Assay System (#MK400, Takara, Japan) was used. The WST‐1 solution was diluted in serum‐free RPMI at 10:1, following the manufacturer's instructions. The absorbance was measured using a microplate reader (SpectraMax iD3; Molecular Devices, USA).

### 5.8 H&E Staining

The printed samples were fixed for 3 days and washed with PBS for 15 min. The fixed spheroids were immersed in 10%, 20%, and 30% sucrose solution for 5 min each at 4°C and left in the solution overnight. Subsequently, the spheroids were centrally placed in a plastic mold and surrounded with OCT compound, and then stored in a deep freezer. For sectioning, the spheroids were cut at a thickness of 8 µm under conditions of −30°C using a cryostat. The sectioned spheroids were placed on a histobond slide and washed with PBS for 5 min to prepare for H&E staining. Hematoxylin was applied for 10 min, followed by a rinse with 1% acidic alcohol and distilled water. Eosin was applied for 10 min and rinsed off with distilled water. The samples were dehydrated through 90% and 100% ethanol solutions and dipped in Histo‐Clear for 10 min. The slides were cleaned with a wipe, and cover glasses were mounted.

### 5.9 Immune Cell Co‐Culture and Quantification of NK Cell Accumulation in a 3D Printing Model

For all immune‐tumor interaction‐related assays, a cell density of 1 × 10^7^/mL in bioink was used (Data S2a,b). After bath printing, HLS was cultured for 3 days in culture media. EGFR CAR‐NK was seeded at 2.5 × 10^4^ per 10 µL of RPMI medium in each HLS and co‐cultured for 16 and 24 h. The used cell density was adjusted to an effector to target ratio of 1.5–2:1 (Data S2c,d). NK cell accumulation was quantified by defining a circular region of interest with a 500 µm radius centered on each spheroid. The NK‐positive area within this region was measured to evaluate NK cell enrichment.

### 5.10 Cell Harvesting and Sorting from the 3D TME Model

Ethylenediaminetetraacetic acid (EDTA; #20‐158, Sigma–Aldrich, USA) and collagenase (#17100017, Gibco) were used to prepare a single‐cell suspension. The alginate bath was degraded using 8 mm EDTA by incubating for 30 min. HLS and NK cells were collected by centrifugation. The cell spheroids and NK cells were treated with 20 mm collagenase for 30 min to dissociate them into single cells.

CD56 microbeads, human (#130‐050‐401, Miltenyi, Germany), were used to separate the tumor components and CAR‐NK cells. The NK cells were labeled using microbeads and magnetically separated using the MACS Separator with the MACS Column, following the manufacturer's instructions.

### 5.11 Flow Cytometry

For apoptosis analysis, the separated cancer cells were washed with fluorescence‐activated cell sorting (FACS) buffer, which is composed of PBS supplemented with 0.1% FBS and 2 mm EDTA, and centrifuged. Annexin V conjugated with APC and 7‐AAD was added to the cell pellets in a 100‐µL binding buffer at room temperature for 15 min (#62700‐80, Biogems, USA). After the reaction, the binding buffer was added according to the cell number. For surface staining, the following antibodies were used: CD56‐BV (#5562751, BD Biosciences, USA), PD‐1‐APC (#558694), NKG2D‐PE (#557940), NKp30‐PE (#558407), NKp44‐PE (#FAB22491P, R&D Systems, USA), NKp46‐PE (#FAB1850P), KIR2DL1‐PE (#FAB1844P), KIR2DL2/3‐PE (#FAB1848P), Myc‐PE (#3739S, Cell Signaling Technology, USA). CXCR6‐BV (#566007, Biolegend, USA), and CXCL16‐BV (#750901). A staining solution with the antibodies was added to the centrifuged cell pellet for 30 min and analyzed by flow cytometry. Flow cytometric analysis was performed using a BD FACS Canto II cytometer (BD Biosciences, USA), and the data were analyzed using FlowJo software (BD Biosciences, USA).

### 5.12 In Vitro Cytotoxicity

The cytotoxicity was conducted using a calcein‐AM‐based assay. The target cells were stained with calcein‐AM for 1 h in an incubator. The stained target cells (1 × 10^4^ cells/100 µL) were washed with RPMI1640 and placed with NK cells (1 × 10^4^ cells/100 µL) in 96‐well plates. Triton X‐100 (4%) was added to the target cells, and the maximum release was measured. The cytotoxicity was calculated using the following equation:

$$\begin{array}{ccc} & & (\mathrm{sample}\;\mathrm{release}-\mathrm{spontaneous}\;\mathrm{release})/(\mathrm{maximum}\;\mathrm{release}\\ & & \;-\;\mathrm{spontaneous}\;\mathrm{release})\;\times \;100 \end{array}$$

### 5.13 RNA Extraction and Real‐Time Quantitative PCR (RT‐qPCR)

Total RNA was extracted using an RNA extraction kit (#74104, QUIAGEN, Germany) following the manufacturer's instructions. The separated cells were used for the RT‐qPCR of the tumor and NK cells. RNA was extracted, and complementary deoxyribonucleic acid (cDNA) was synthesized using a cDNA synthesis kit (#PSK‐101, Toyobo, Japan). Real‐time PCR was used with a Thermal Cycler Dice TP800 and a qPCR Master Mix (#70201SG, Addbio, South Korea). All data were normalized to the housekeeping gene GAPDH. The primer sequences are listed in Table S1.

### 5.14 Cytokine Release Assay

Cytokine secretion was quantified using an enzyme‐linked immunosorbent assay (ELISA). For NK functional assays, CAR‐NK92 cells were co‐cultured with 3D HLS tumor models of the indicated complexity for 24 h. For CXCL16 analysis, NSCLC cell lines (H460, A549, PC‐9, and H1650) were seeded at equal densities and cultured for 4 days under standard conditions.

Following incubation, culture supernatants were collected and clarified by centrifugation (300 × g, 10 min) to remove cells and debris. The clarified supernatants were analyzed by ELISA for IFN‐γ (#88‐7316‐88, Thermo Fisher Scientific), TNF‐α (#88‐7346‐88, Thermo Fisher Scientific), and CXCL16 (#900‐K230K, Peprotech). All ELISA assays were performed in accordance with the manufacturer's instructions.

### 5.15 mRNA In Vitro Transcription and Transfection

mRNA‐encoded CXCR6 was generated by synthesizing mRNA using the in vitro transcription kit (#AM1345, Invitrogen). As a negative control, control mRNA was synthesized from the control template included in the same kit and used to match electroporation and total mRNA input. NK92 or CAR‐NK92 cells (2 × 10^6^) were transfected with indicated amounts of CXCR6 mRNA or an equal amount of control mRNA in 100 µL Opti‐MEM. After 8 h, the cells were analyzed using flow cytometry for CXCR6 expression levels. Electroporation was carried out using the NEPA21 electroporator (Nepa Gene, Japan) following the guidelines provided by the manufacturer.

### 5.16 Transwell Migration Assay

To assess migration, CXCR6‐expressing or control mRNA–transfected NK or CAR‐NK cells were suspended in serum‐free RPMI 1640 medium at a concentration of 4 × 10^5^ cells per 200 µL and loaded into the upper chamber of a transwell insert (#3421, Corning, USA). The lower chamber was filled with 600 µL of RPMI 1640 medium containing 10% FBS and 50 ng/mL CXCL16. The transwell plates were incubated at 37°C in a CO_2_ incubator for 4 h. After incubation, cells that had migrated to the lower chamber were collected and counted using trypan blue staining.

The chemotaxis index was calculated as the fold increase in migrated cells relative to the control mRNA–transfected NK cell group. All experimental groups were assessed under identical serum gradient conditions.

### 5.17 Western Blot

Cells were transfected with CXCR6 mRNA and, at various time points, treated with CXCL16 (50 ng/mL) for 30 min before preparation. For protein extraction, the cells were lysed using a protein lysis buffer (#4719956001, Roche, Switzerland) supplemented with protease and phosphatase inhibitors (#4906837001, Roche). The protein concentrations were measured using the Pierce BCA Protein Assay Kit (#23225, Thermo Fisher Scientific). Equal amounts of protein (10–20 µg) from the lysates were loaded onto 12% SDS‐PAGE gels and transferred onto 0.45 µm PVDF membranes (#IPVH00010, Merck Millipore, Germany). The membranes were then blocked with 5% skim milk at room temperature for 40–60 min and incubated overnight at 4°C with the following primary antibodies: GAPDH (#5174, Cell Signaling Technology), pERK (#9101), ERK (#9102), pAKT (#9275), and AKT (#9272). After washing, the membranes were incubated with HRP‐conjugated anti‐rabbit IgG (#31460, Thermo Fisher Scientific). Protein signals were developed using the SuperSignal West Pico Chemiluminescent Substrate (#34078, Thermo Fisher Scientific) and visualized with the WSE‐6100 LuminoGraph (ATTO, USA).

### 5.18 Preparation and Processing for RNA Sequencing

The cell was prepared through a cell harvesting process using EDTA and collagenase. The total RNA was extracted and submitted to Macrogen (Seoul, Korea) for mRNA sequencing and library preparation.

RNA‐seq with paired‐end reads was performed using the NovaSeq X platform (Illumina, CA, USA). Low‐quality reads were filtered out, and the resulting clean reads were mapped to the human transcriptome reference obtained from the Ensembl genome browser (assembly ID: GRCh38). The HISAT2 software (ver. 2.2.0) was used to index the reference genome, while the featureCounts (ver. 2.0.1) software was used to calculate the counts of mapped reads aligning to genomic features from the binary sequence alignment (BSM) files generated using SAMtools (ver. 1.15.1) [74, 75]. The count data were normalized to fragments per kilobase of transcript per million mapped reads (FPKM) values, and a log2 transformation was performed.

### 5.19 Public Gene Expression Profile Data

The clinical information and gene expression profile data on lung cancer patients provided by The Cancer Genome Atlas Program (TCGA; n = 555) were downloaded from the Genomic Data Commons (GDC) data portal (https://portal.gdc.cancer.gov) [76]. Other gene expression profiles from 2D and Matrigel‐based 3D cultures of A549 cells were obtained from the Gene Expression Omnibus (GEO) database (GSE130590; https://www.ncbi.nlm.nih.gov/geo). Although this dataset has not been associated with a peer‐reviewed publication, it was deposited and made publicly available in 2019 by the submitter. All public expression profiles were processed using the FPKM value.

### 5.20 Transcriptomic Analysis

We selected 731 significantly differentially expressed genes (fold change ≥ 1.5) between the HLS model and the average of the other two models. To explore enriched biological processes, we performed a DAVID functional annotation bioinformatics microarray analysis using the Gene Ontology (GO) biological process category, identifying significant processes based on a False Discovery Rate (FDR) threshold of < 0.01. To establish a validation gene set specifically representing the HLS model, we further refined the 731 DEGs by excluding genes inherently highly expressed in fibroblasts. This was achieved by comparing the gene expression profiles of the human lung fibroblast cell line (HLFA_FIBROBLAST) and the A549 non‐small cell lung cancer cell line using the Cancer Cell Line Encyclopedia (CCLE) database (https://sites.broadinstitute.org/ccle/) [77]. Genes with a fold change of < 1.5 between these two cell lines were selected, resulting in a final validation gene set comprising 182 genes highly expressed in the HLS model and 102 genes with lower expression. This gene signature is provided in the Data File. To quantify the model‐specific characteristics, we calculated the HLS model‐associated score by standardizing the expression of each gene and subtracting the average expression of the lower‐expressed genes from the average of the highly‐expressed genes. The t‐distributed stochastic neighbor embedding (t‐SNE) plot was generated based on the gene expression profiles of this validation gene set to visualize the distinct clustering of the HLS model. The expression levels of all samples, including the three models and patient data, were standardized before analysis. The perplexity parameter was set to 30, and the plot was embedded in two dimensions. The distribution patterns were visualized as dot plots. Correlation analysis was performed by calculating the average correlation between each model and individual lung cancer patient expression profiles. Before the calculation, genes with a standard deviation greater than 0.8 across the TCGA lung cancer dataset were selected (*n* = 5,403), and all gene expression profiles were standardized. All transcriptomic analyses were performed using R software (version 4.2.1).

### 5.21 Statistical Analysis

All results are presented as mean ± s.d. Statistical analysis was performed using GraphPad Prism v6 software (GraphPad Software Inc., USA). One or two‐way ANOVA, followed by Tukey's post hoc test, was performed for multiple‐group analyses, and unpaired two‐tailed Student t‐tests were performed for two‐group analyses. The survival analysis was assessed using the log‐rank test. The degree of significance is indicated as follows: N.S. (not significant) = *p* > 0.05, ^∗^ *p* < 0.05; ^∗∗^ *p* < 0.01; and ^∗∗∗^ *p* < 0.001.

## Author Contributions

D.K. and S.J. contributed equally to this work. D.K. and S.J. conceived and designed the study, performed and analyzed experiments, and wrote the manuscript. I.H.J. performed transcriptomic analysis and wrote the manuscript. Y.J.K., Y.J., and J.A. provided essential materials. H.L., J.J.L., and K.L. designed the experiments. T.D.K. and S.A.P. designed and supervised the study, acquired funding, and revised the manuscript.

## Conflicts of Interest

The authors declare no conflicts of interest.

## Supporting information




**Supporting File 1**: advs75140‐sup‐0001‐SuppMat.pdf.

**Supporting File 2**: advs75140‐sup‐0002‐VideoS1.avi.

**Supporting File 3**: advs75140‐sup‐0003‐DataFile.xlsx.

## Data Availability

The complete mRNA sequencing data have been deposited in the GEO database and can be accessed under the number (GEO: GSE298447).
