# Supplementary material for: Bioprinted Tumor Microenvironment Models Reveal Immune Evasion and Guide CAR‐NK Therapeutic Strategies
Source: Adv Sci (Weinh). 2026 May 8;13(36):e21188. doi: 10.1002/advs.202521188 (PMC13317579; doi:10.1002/advs.202521188)
Supplement: Supplementary file 1 — Supporting File 1: advs75140‐sup‐0001‐SuppMat.pdf. [file ADVS-13-e21188-s002.pdf]

Supporting Information

Dahong Kim[a,b†], Seona Jo[c,d†], In-Hwan Jang[c], Yu-Jin Kim[f], Youngmee Jung[f], Junhyoung Ahn[g], Hyungjun Lim[a], Jae Jong Lee[a], Kangwon Lee[b], Tae-Don Kim[c,d,e], Su A Park[a*]*

Dahong Kim, Junhyoung Ahn, Hyungjun Lim, Jae Jong Lee, Su A Park
[a]Nano Lithography & Manufacturing Research Center, Nano-Convergence Manufacturing Research Division, Korea Institute of Machinery and Materials (KIMM),
Daejeon, 34103, Republic of Korea

Dahong Kim, Kangwon Lee
[b]Department of Applied Bioengineering Graduate School of Convergence Science and Technology, Seoul National University, Seoul, 08826, Republic of Korea

Seona Jo, In-Hwan Jang, Tae-Don Kim
[c]Center for Gene and Cell Therapy, Korea Research Institute of Bioscience and Biotechnology (KRIBB), Daejeon, Republic of Korea

Seona Jo, Tae-Don Kim
[d]KRIBB School of Advanced Bioconvergence, University of Science and Technology (UST), Daejeon, Republic of Korea

Tae-Don Kim
[e]Department of Pharmacy, Yonsei Institute of Pharmaceutical Sciences , Department of Integrative Biotechnology, College of Pharmacy, Yonsei University, Incheon 21983, Republic of Korea

Yu-Jin Kim, Youngmee Jung
[f]Center for Biomaterials, Korea Institute of Science and Technology, Seoul, 02792, Republic of Korea

Youngmee Jung
[g]Department of Electrical and Electronic Engineering, YU-KIST Institute, Yonsei University, Seoul, 03722, Republic of Korea

Figure S1

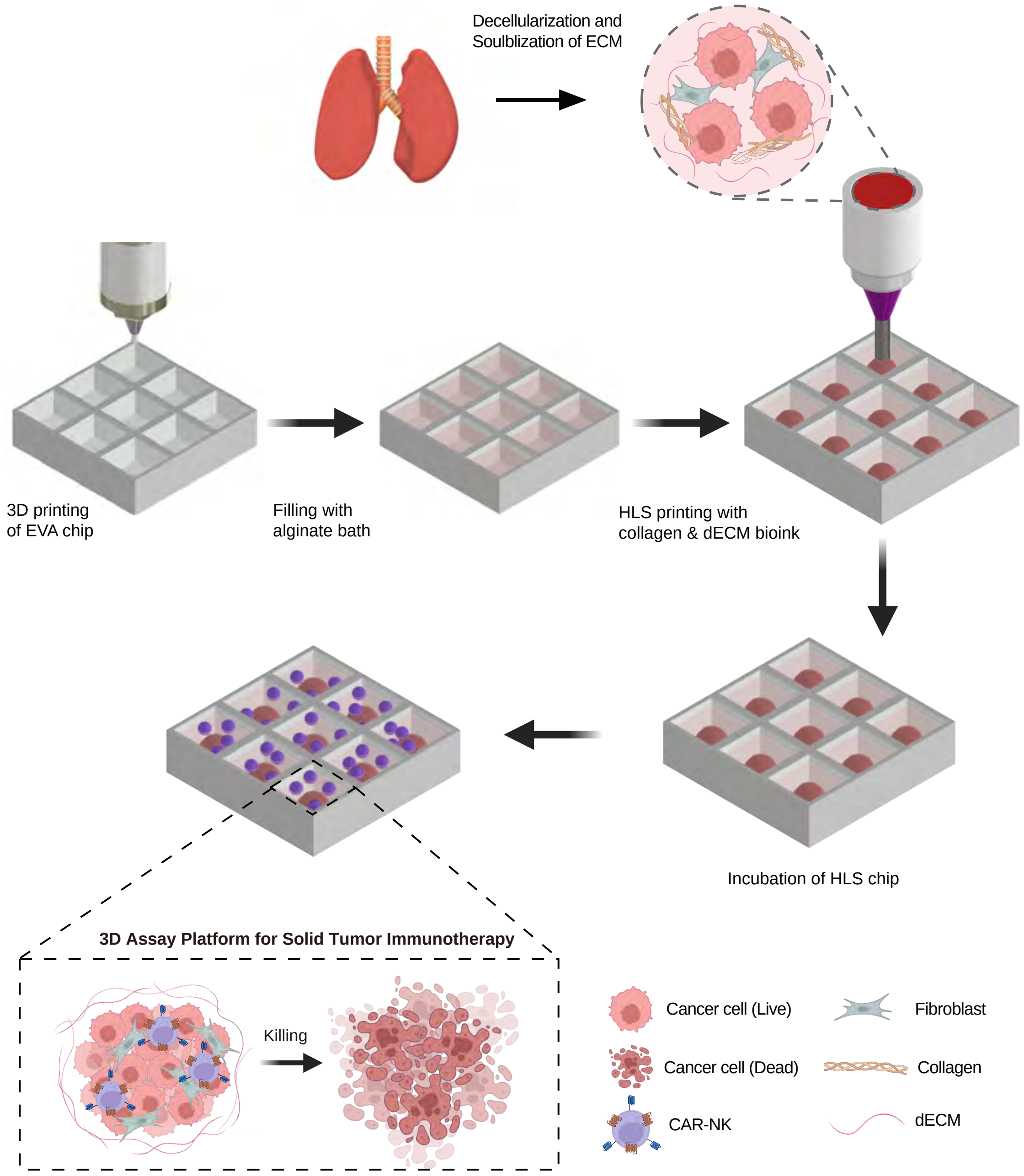

Figure S1. Schematic Illustration of 3D Embedded Bioprinting Process

Diagram detailing the steps involved in the embedded bioprinting process, including the printing of an EVA chip, filling it with a 1.5% alginate bath, depositing HLS using bioink, and conducting tests for immune-cancer interactions.

Figure S2

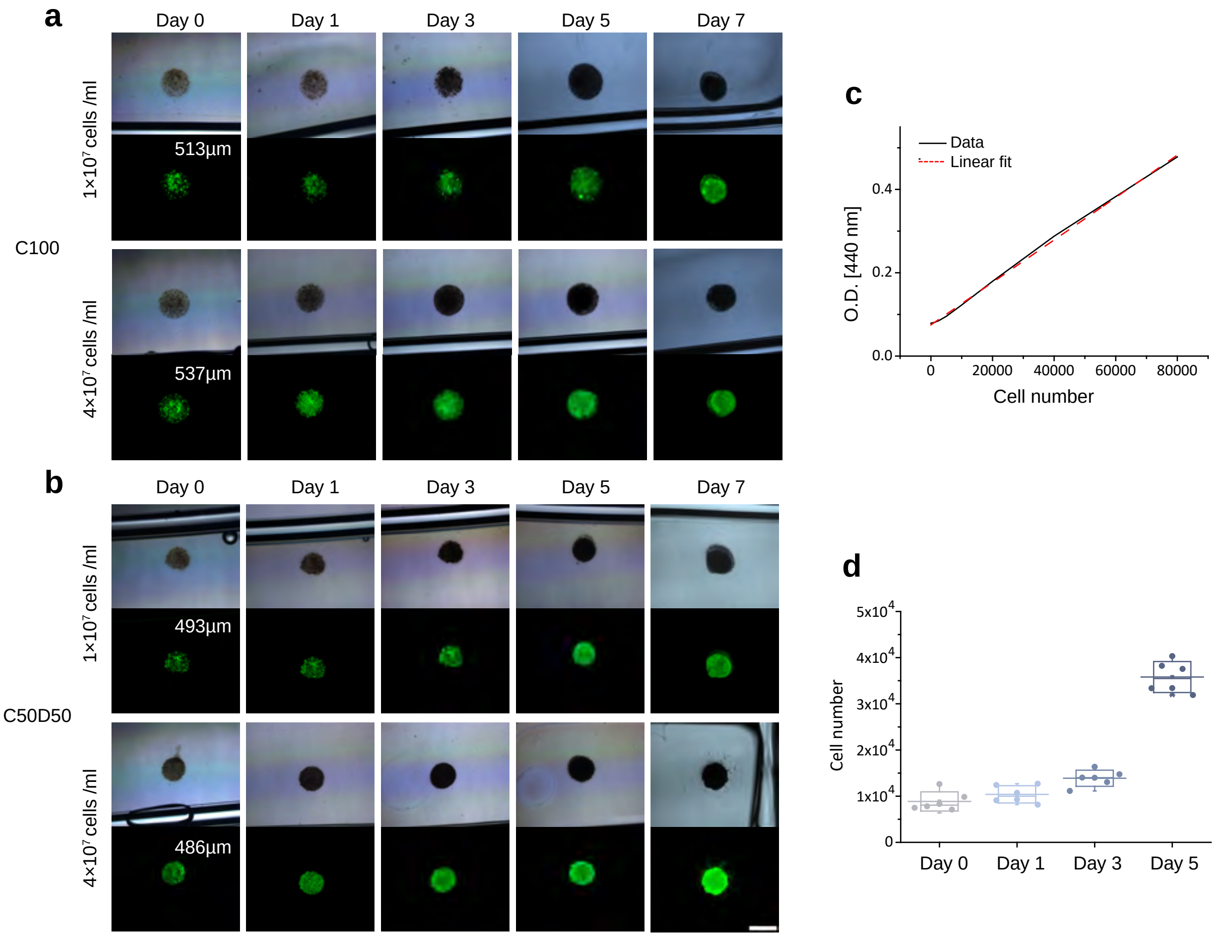

Figure S2. Cell Viability and Cell Number with Different Cell Concentration

a. Representative fluorescent images of live and dead assay for varying cell concentration in C100 bio-ink and b. C50D50. Red: dead cells; Green: live cells; scale bar: 500 µm. c. Linear fit graph depicting the relationship between optical density (O.D) intensity and cell number. d. Quantification of cell numbers within the bio-ink. d. Quantification of cell number in bio-ink. Data are mean ± s.d, with the interquartile range from the 25 to 75 percentile. (n=6).

Figure S3

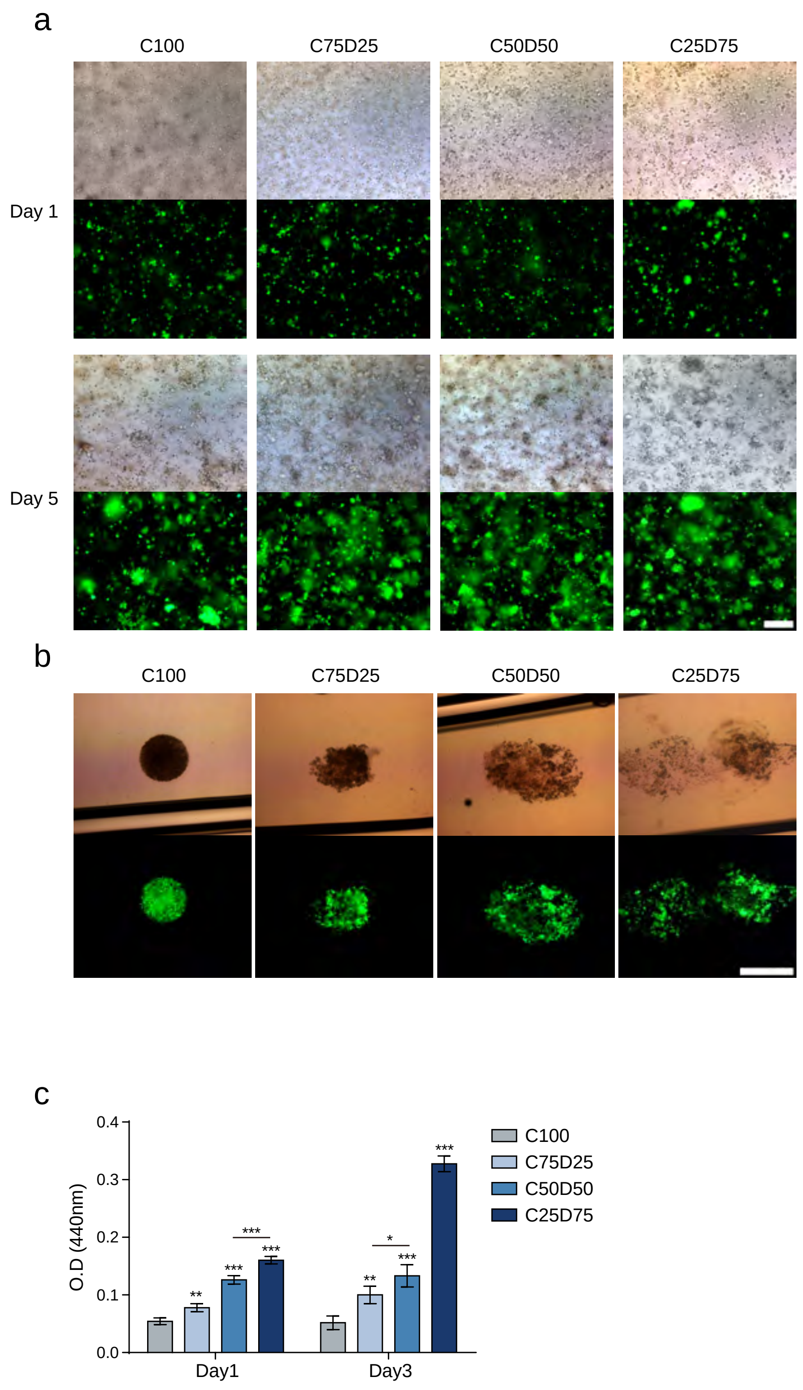

Figure S3. Cell Viability and Printability of Collagen and 1% dECM according to Ratio

a. Representative fluorescent images of live and dead assays for A549 cell line encapsulated in bio-ink over 1 to 5 days; Red: dead cells; Green: live cells; scale bar: 500 μm. b. Representative fluorescent images demonstrating the printability of bio-ink composed of collagen and 1% dECM. c. Quantification of cell proliferation in bio-ink with collagen and 1% dECM. Data are mean ± s.d. (n=4).

Figure S4

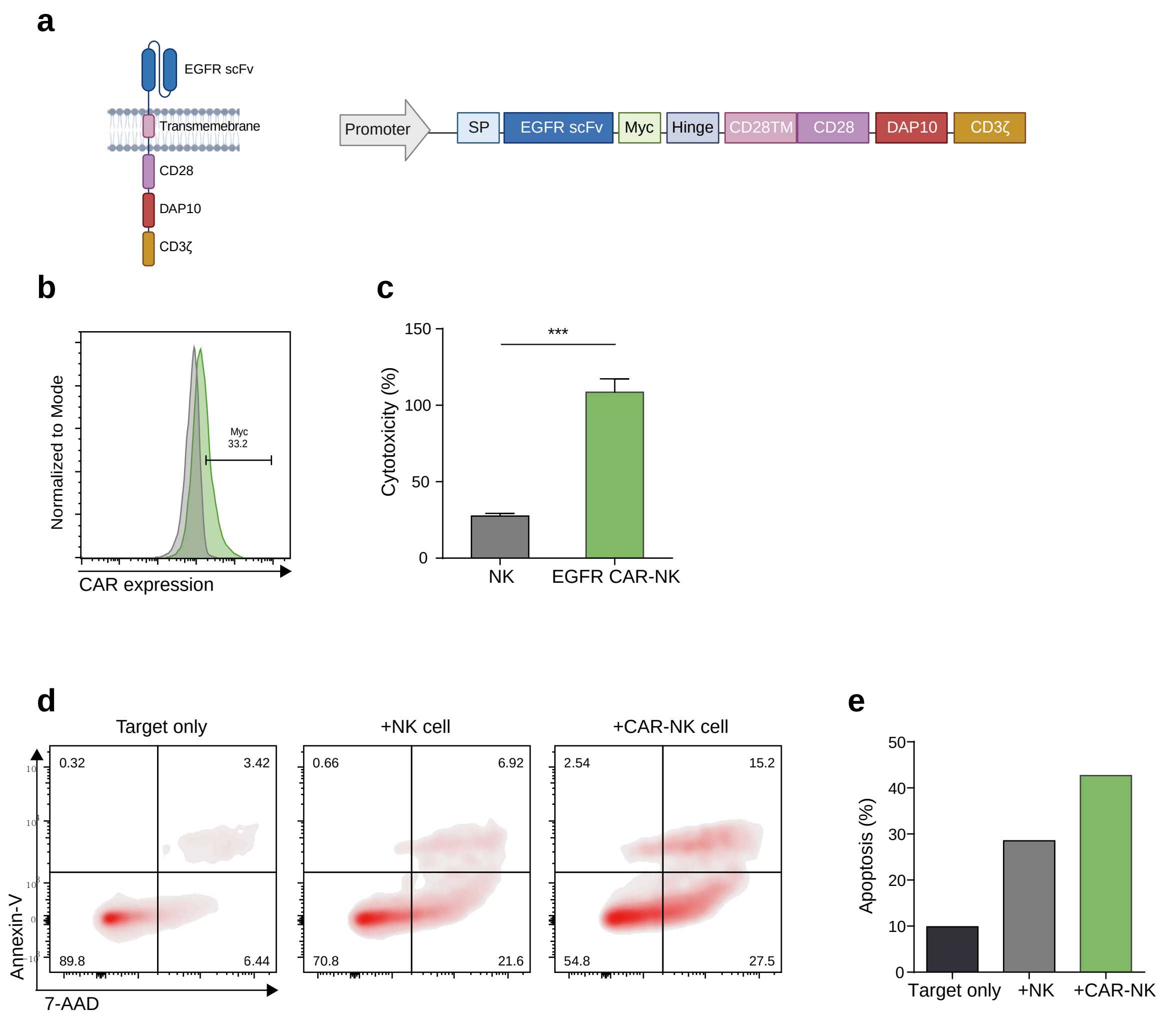

Figure S4. EGFR CAR-NK Anti-Cancer Activity in 2D-cultured

a. Diagram of the third-generation EGFR CAR structure, including EGFR scFv, a myc tag, a hinge region, CD28 (transmembrane to intracellular domain), DAP10, and CD3ζ. b. Flow cytometry analysis of CAR expression on NK92 cells. Anti-myc staining of untransduced NK92 cells served as a negative control. c. Cytotoxicity of EGFR CAR-NK cells co-incubated with A549 cells at an effector-to-target (E:T) ratio of 5:1 for 4 hours. Data are mean ± s.d. (n=3) Statistical significance was determined using unpaired two tailed student t-tests. d. Apoptosis of A549 cells co-incubated with EGFR CAR-NK cells for 4 hours, detected by 7-AAD and Annexin-V staining using flow cytometry. e. Percentage of apoptosis represented by Annexin-V-positive A549 cells.

Figure S5

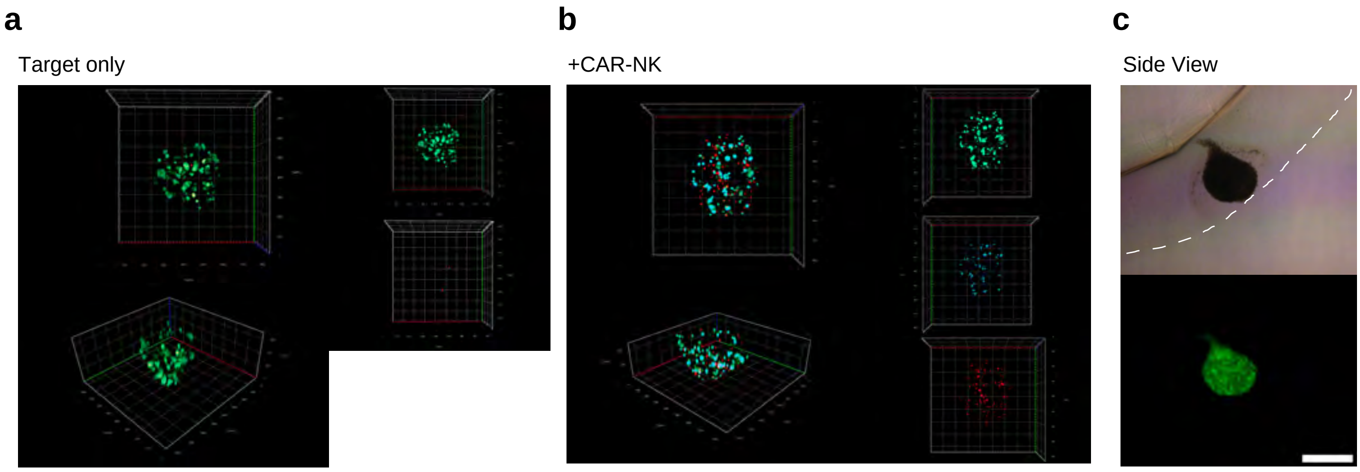

Figure S5. 3D Confocal Imaging of HLS Spheroids with and without CAR-NK Cells

a. Representative 3D confocal image of an HLS spheroid without CAR-NK cells showing target cell viability. Red indicates dead cells; green indicates live cells; blue marks CAR-NK cells. b. Similar image with CAR-NK cells.

c. Representative fluorescent images of the side view of the HLS spheroid.

Figure S6

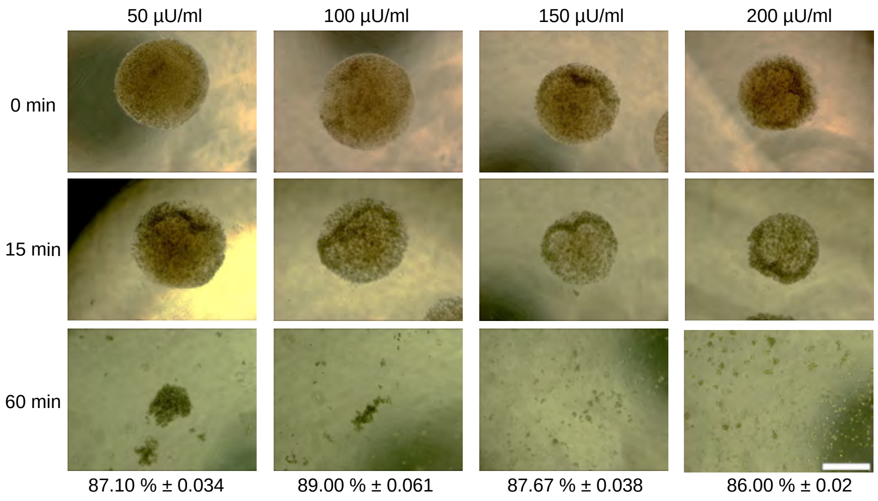

Figure S6. Effects of Collagenase Treatment on Spheroid Dissociation and Cell Viability

Representative microscopic image showing the disassociation of spheroids and cell viability following treatment with different concentrations of collagenase (50, 100, 150, and 200 µU/ml) over time.

Figure S7

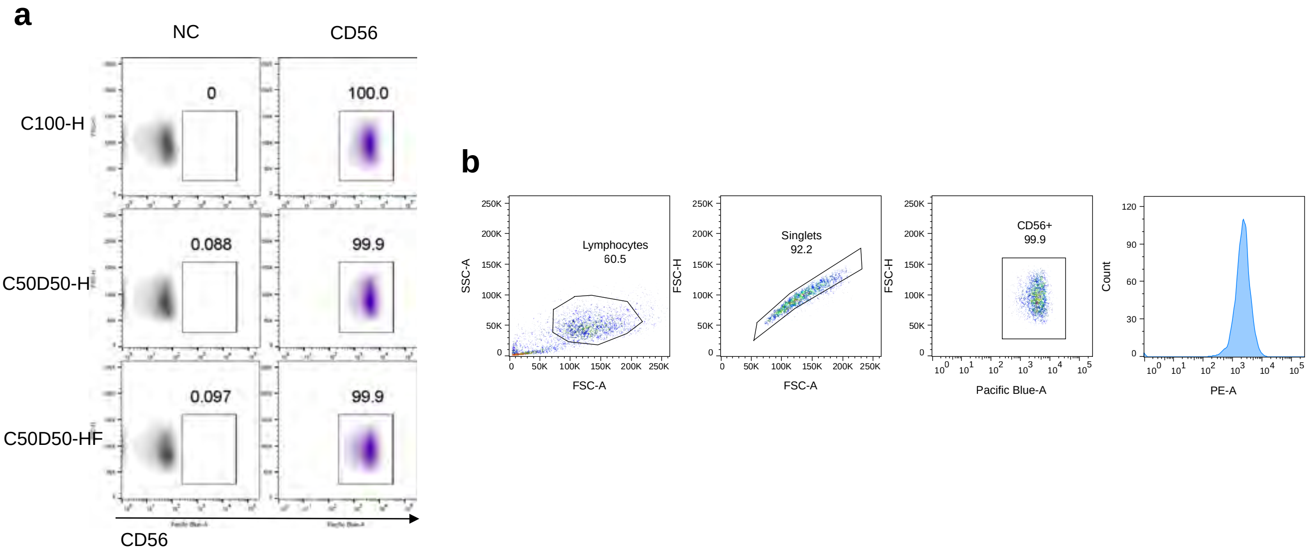

Figure S7. CD56 Expression After Magnetic Bead Isolation in 3D-cultured Samples

a. CD56 expression detected by flow cytometry following CD56 magnetic bead isolation.

b. Representative gating strategy used for NK receptor analyses.

Figure S8

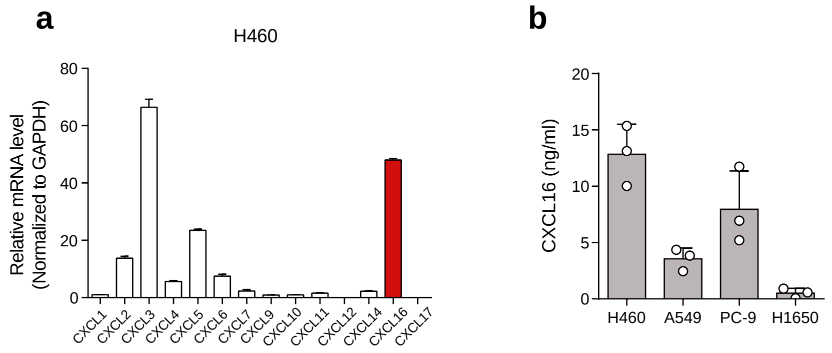

Figure S8. Lung Tumor Express CXCL16

a. Screening of CXC-motif chemokine mRNA expression in H460 cells by qRT-PCR. b. CXCL16 release measured ELISA by from lung cancer cell line-conditioned supernatant after 4 days. Data are mean ± s.d. (n=3).

Figure S9

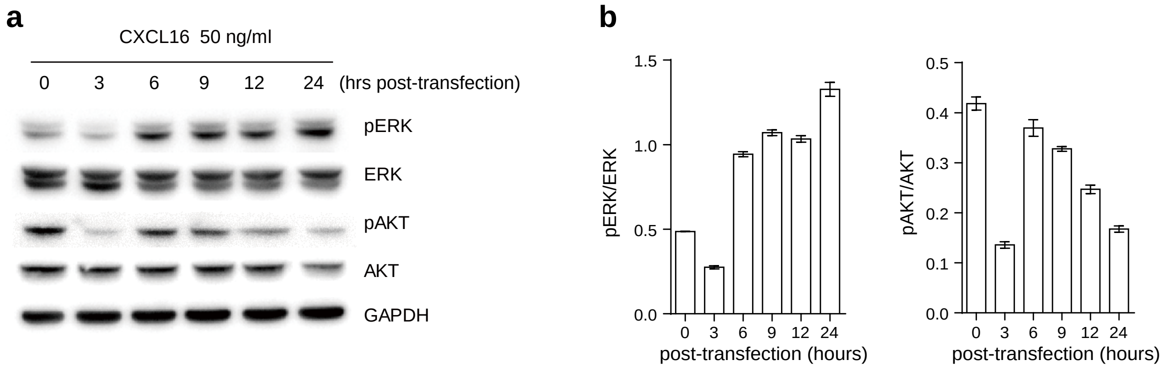

Figure S9. Lung Tumor Express CXCL16

a. Intracellular signaling analyzed by Western blot after treating CXCR6 mRNA-transfected cells with 50 ng/μL CXCL16 for 30 minutes at various time points post-transfection (0, 3, 5, 6, 12, 24 hours). b. Densitometric quantification of p-ERK/ERK and p-AKT/AKT ratios normalized to total protein levels. Data are mean ± s.d. (n=3).

Figure S10

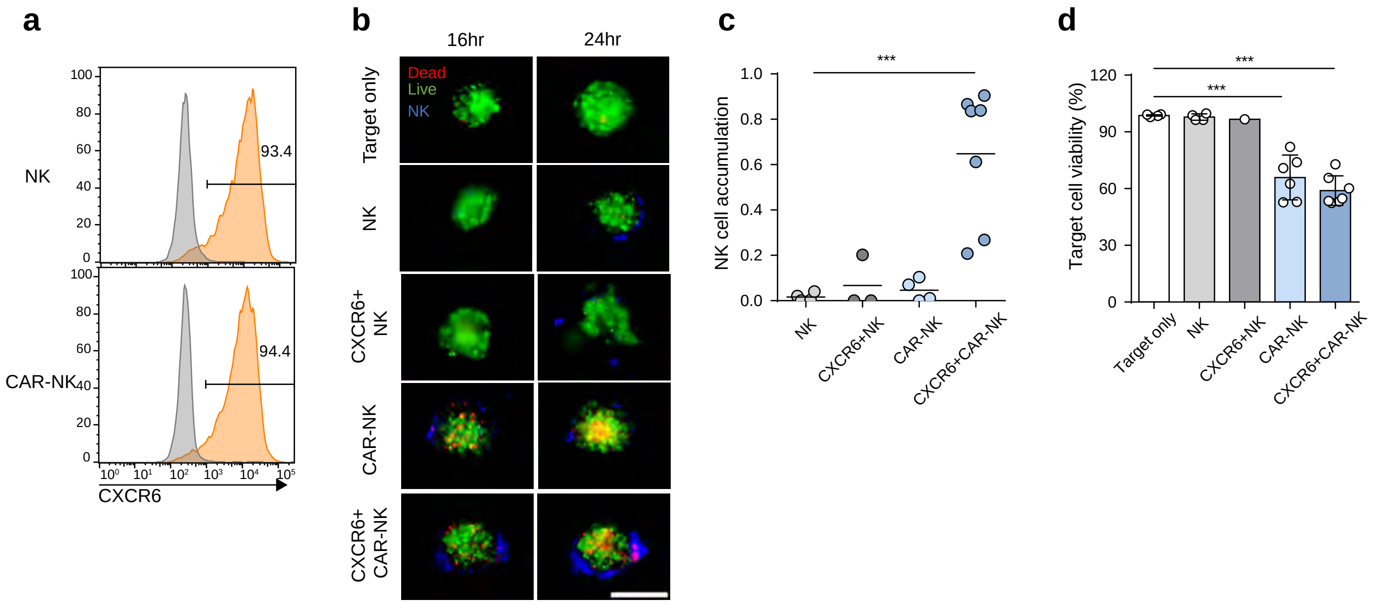

Figure S9. 3D-Printed HLS Model Without dECM Co-Cultured with CXCR6 Overexpressing NK and CAR-NK Cells

a. Representative fluorescent images of NK or CAR-NK cells co-cultured in the 3D printed HLS without dECM. Red: dead cells; Green: live cells; Blue: NK or CAR-NK cells. b. NK cell accumulation near target cells in the HLS model, quantified as the NK cell area/total cell area ratio c. Viability analysis of target cells after 24 hours of co-culture. Data are mean ± s.d. (n=3-6) Statistical significance was determined using two-way ANOVA.

Supplementary table

Target gene	Primer probe	Sequence (5'→ 3')
MMP1	Forward	ACAAACCCCAAAAGCGTGTG
	Reverse	AGAAGGGATTTGTGCGCATG
MMP2	Forward	CCCTGTGTCTTCCCCTTCAC
	Reverse	GGCCTCGTATACCGCATCAA
S100A9	Forward	GCAAAATGTCGCAGCTGGAA
	Reverse	CTTATGGTGGTGGCCAGGG
VEGF	Forward	TTGCCTTGCTGCTCTACCTC
	Reverse	AAATGCTTTCTCCGCTCTGA
ITGA5	Forward	TCAACTGCACCACCAATCAC
	Reverse	TTGGAGCTTCCCGTTTTTGC
E-CAD	Forward	ACCTGAACGACTGGGGGCCA
	Reverse	TGCCAAAGCCTCCAGCAAGCA
TGF-β1	Forward	CAAGCAGAGTACACACAGCAT
	Reverse	TGCTCCACTTTTAACTTGAGCC
IL-6	Forward	AGTCCTGATCCAGTTCCTGC
	Reverse	CTACATTTGCCGAAGAGCCC
PD-L1	Forward	CACGGTTCCCAAGGACCTAT
	Reverse	GGCCCTCTGTCTGTAGCTAC
PD-L2	Forward	TGGCAGAAACTTCAGCTGTG
	Reverse	ATGTGAAGCAGCCAAGTTGG
NKp30	Forward	TGAGATTCGTACCCTGGAAGG
	Reverse	CACTCTGCACACGTAGATGCT
CD107a	Forward	ACACACCTTTTCCCCAATGC
	Reverse	ACGTTGTTCATGTGGACCTG
Perforin	Forward	TGTAACCAGGGCCAAAGTCAG
	Reverse	ACACATGCACATTGCTGGTG
Granzyme B	Forward	CCCTGGGAAAACACTCACACA
	Reverse	CACAACTCAATGGTACTGTCGT
TNF-α	Forward	AGGACCAGCTAAGAGGGAGA
	Reverse	CCCGGATCATGCTTTCAGTG
GM-CSF	Forward	GGAGCATGTGAATGCCATCCAG
	Reverse	CTGGAGGTCAAACATTTCTGAGAT
BIM	Forward	GGTCCTCCAGTGGGTATTTCTC
	Reverse	ACTGAGATAGTGGTTGAAGGCCTGG
Bcl-2	Forward	ATGTGTGTGGAGAGCGTCAA
	Reverse	ACAGTTCCACAAAGGCATCC
HIF1-α	Forward	ATGATGTAATGCTCCCCTCACC
	Reverse	AAGTGGCTTTGGCGTTTCAG
GAPDH	Forward	GAGTCAACGGATTTGGTCGT
	Reverse	TTGATTTTGGAGGGATCTCG

Target gene	Primer probe	Sequence (5'→ 3')
CXCL1	Forward	CTGCTCCTGCTCCTGGTAG
	Reverse	ATGCAGGATTGAGGCAAGCT
CXCL2	Forward	GCAGGGAATTCACCTCAAGAAC
	Reverse	TTCTTAACCATGGGCGATGC
CXCL3	Forward	ACACTGCAGGGAATTCACCTC
	Reverse	GTGTGGCTATGACTTCGGTTTG
CXCL4	Forward	GCGCTGAAGCTGAAGAAGATG
	Reverse	TCCATTCTTCAGCGTGGCTATC
CXCL5	Forward	GCTGCGTTGCGTTTGTTTAC
	Reverse	GGCGAACACTTGCAGATTACTG
CXCL6	Forward	GCTGCGTTGCACTTGTTTAC
	Reverse	ACTTGCTTCCCGTTCTTCAG
CXCL7	Forward	TGCTGCTTCTGTCATTGCTG
	Reverse	ATACACATGCAGCGGAGTTC
CXCL9	Forward	TAGTGAGAAAGGGTCGCTGTTC
	Reverse	GAAGGGCTTGGGGCAAATTG
CXCL10	Forward	ACCTCTCTCTAGAACTGTACGC
	Reverse	CGTGGACAAAATTGGCTTGC
CXCL11	Forward	CCTGGGGTAAAAGCAGTGAAAG
	Reverse	GCCTTGCTTGCTTCGATTTG
CXCL12	Forward	TCTTCGAAAGCCATGTTGCC
	Reverse	TTCGGGTCAATGCACACTTG
CXCL14	Forward	GGTTATCATCACCACCAAGAGC
	Reverse	ACTTGATGAAGCGCTTGGTG
CXCL16	Forward	TGGCCATCATCTTCATCCTCAC
	Reverse	TAATGAACCGGCAGATCTGGAG
CXCL17	Forward	TGTTGCTGCCACTAATGCTG
	Reverse	GCTCTCAGGAACCAATCTTTGC
